# Novel Fungal Pathogenicity and Leaf Defense Strategies Are Revealed by Simultaneous Transcriptome Analysis of *Colletotrichum fructicola* and Strawberry Infected by This Fungus

**DOI:** 10.3389/fpls.2018.00434

**Published:** 2018-04-25

**Authors:** Liqing Zhang, Xin Huang, Chengyong He, Qing-Yu Zhang, Xiaohua Zou, Ke Duan, Qinghua Gao

**Affiliations:** ^1^Shanghai Key Laboratory of Protected Horticultural Technology, Forestry and Fruit Tree Research Institute, Shanghai Academy of Agricultural Sciences, Shanghai, China; ^2^College of Food Science, Shanghai Ocean University, Shanghai, China

**Keywords:** *Colletotrichum fructicola*, strawberry, effector, pathogenicity, RNA-seq, resistance

## Abstract

*Colletotrichum fructicola*, which is part of the *C. gloeosporioides* species complex, can cause anthracnose diseases in strawberries worldwide. However, the molecular interactions between *C. fructicola* and strawberry are largely unknown. A deep RNA-sequencing approach was applied to gain insights into the pathogenicity mechanisms of *C. fructicola* and the defense response of strawberry plants at different stages of infection. The transcriptome data showed stage-specific transcription accompanied by a step-by-step strawberry defense response and the evasion of this defense system by fungus. Fungal genes involved in plant cell wall degradation, secondary metabolism, and detoxification were up-regulated at different stage of infection. Most importantly, *C. fructicola* infection was accompanied by a large number of highly expressed effectors. Four new identified effectors function in the suppression of Bax-mediated programmed cell death. Strawberry utilizes pathogen-associated molecular patterns (PAMP)-triggered immunity and effector-triggered immunity to prevent *C. fructicola* invasion, followed by the initiation of downstream innate immunity. The up-regulation of genes related to salicylic acid provided evidence that salicylic acid signaling may serve as the core defense signaling mechanism, while jasmonic acid and ethylene pathways were largely inhibited by *C. fructicola*. The necrotrophic stage displayed a significant up-regulation of genes involved in reactive oxygen species activation. Collectively, the transcriptomic data of both *C. fructicola* and strawberry shows that even though plants build a multilayered defense against infection, *C. fructicola* employs a series of escape or antagonizing mechanisms to successfully infect host cells.

## Introduction

Anthracnose caused by *Colletotrichum* spp. is a devastating disease of cultivated strawberry (*Fragaria* × ananassa Duch) (Henz et al., 1992; Xiao et al., 2004). The *C. gloeosporioides* species complex is the most prevalent agent of strawberry anthracnose in China (Xie et al., 2010). *C. gloeosporioides* usually produce a reddish-brown necrosis of crown tissue following infection. Under greenhouse conditions or in summer nurseries, *C. gloeosporioides* may also produce necrosis on stolons, lesions on fruit, or black leaf spots (MacKenzie et al., 2006). *C. fructicola*, a gloeosporioides aggregate formerly known as *C. gloeosporioides*, is responsible for strawberry anthracnose in Korea and Japan (Nam et al., 2013; Gan et al., 2017). *C. fructicola* is also an important causal agent of strawberry anthracnose in Hubei, China. In inoculated strawberry plants, *C. fructicola* showed strong pathogenicity to both the leaves and petioles of strawberries, with mortality occurring in 77.8% of plants 30 days after inoculation (Han et al., 2016). Many *Colletotrichum* spp. utilize a hemibiotrophic infection strategy (O'Connell et al., 2004). Firstly, the pathogen uses a melanized appressorium to penetrate host tissue (Kubo and Takano, 2013). Then, the pathogen produces biotrophic primary hyphae. Within a few days, narrower, secondary hyphae form and proliferate through necrotrophic growth (Perfect et al., 1999; O'Connell et al., 2004).

The interplay between pathogen virulence and host resistance during the co-evolution of fungal plant pathogens and their hosts has been complicated (Koeck et al., 2011). Successful pathogens can dampen basal defenses by producing effectors that inhibit pathogen-associated molecular pattern (PAMP)-triggered immunity (PTI). However, in turn plants can detect such effectors and mount a second layer of defense called effector-triggered immunity (ETI) (Katagiri and Tsuda, 2010). These inducible defenses are also associated with wide-ranging transcriptional and hormonal reprogramming in plants (Pieterse et al., 2012). Phytohormones are a central regulator of immunity. Salicylic acid (SA) is the core plant defense hormone against biotrophic and hemi-biotrophic pathogens; meanwhile, jasmonic acid (JA) is important for resistance to necrotrophic pathogens (Glazebrook, 2005). In the context of basal resistance, cross talk between the SA signaling pathway and other hormone signaling pathways, such as JA, auxin, or abscisic acid (ABA), are often mutually antagonistic (Pieterse et al., 2012).

The interactions between strawberries and *Colletotrichum* spp. have recently been reported (Anwar and Ding, 2004; Li et al., 2013). Anwar and Ding (2004) cloned the strawberry class II chitinase genes, *FaChi2-1* and *FaChi2-2*, and found that both were up-regulated at the mRNA level after *C. fragariae* or *C. acutatum* infection. Using a homologous cloning approach, several nucleotide–binding site–leucine–rich repeat (NBS-LRR) resistance genes were found to be involved in the ecotype-specific responses to *C. gloeosporioides* in strawberries (Li et al., 2013). These preliminary studies provide some information on *Colletotrichum*–strawberry interactions. However, strawberries' resistance to *Colletotrichum* has been reported to be mostly polygenic and quantitatively inherited (Zebrowska et al., 2006; Amil-Ruiz et al., 2011). Therefore, a comprehensive approach is required to fully understand the interaction between the pathogen and the strawberry.

RNA-seq has been used to study *Colletotrichum–*host interactions. O'Connell et al. (2012) investigated *C. higginsianum* and *C. graminicola* at three stages of development in *Arabidopsis thaliana* and maize, respectively. This study revealed that the pathogenicity–related genes of the fungi are successively transcribed and linked to pathogenic transitions (O'Connell et al., 2012). A recent study investigated the expression profiles of mango fruit during *C. gloeosporioides* infection and showed that most of the defense-related genes, such as those encoding ethylene response factors (ERFs), NBS-LRRs, and pathogenesis–related proteins (PRs), were up-regulated after *C. gloeosporioides* infection (Hong et al., 2016). However, these transcriptome studies were focused on either the pathogen or the host; few studies have simultaneously analyzed the response of both the pathogen and the host using RNA-seq. Alkan et al. performed a simultaneous transcriptomic analysis of *C. gloeosporioides* and the tomato plant and demonstrated stage-specific transcription and concurrent changes in fruit response (Alkan et al., 2015). Nevertheless, to date, no comprehensive stage-specific transcriptomic study has been performed to evaluate the interactions between *C. fructicola* and strawberry.

We performed dual RNA-seq profiling to simultaneously determine the transcriptomes of strawberry and *C. fructicola* throughout the infection process. These data provide novel insights into the infection process of *C. fructicola* and the regulation of strawberry defense genes during infection.

## Results and discussion

### Proceeding of *C. fructicola* infection on strawberry leaves

**Figure 1:**
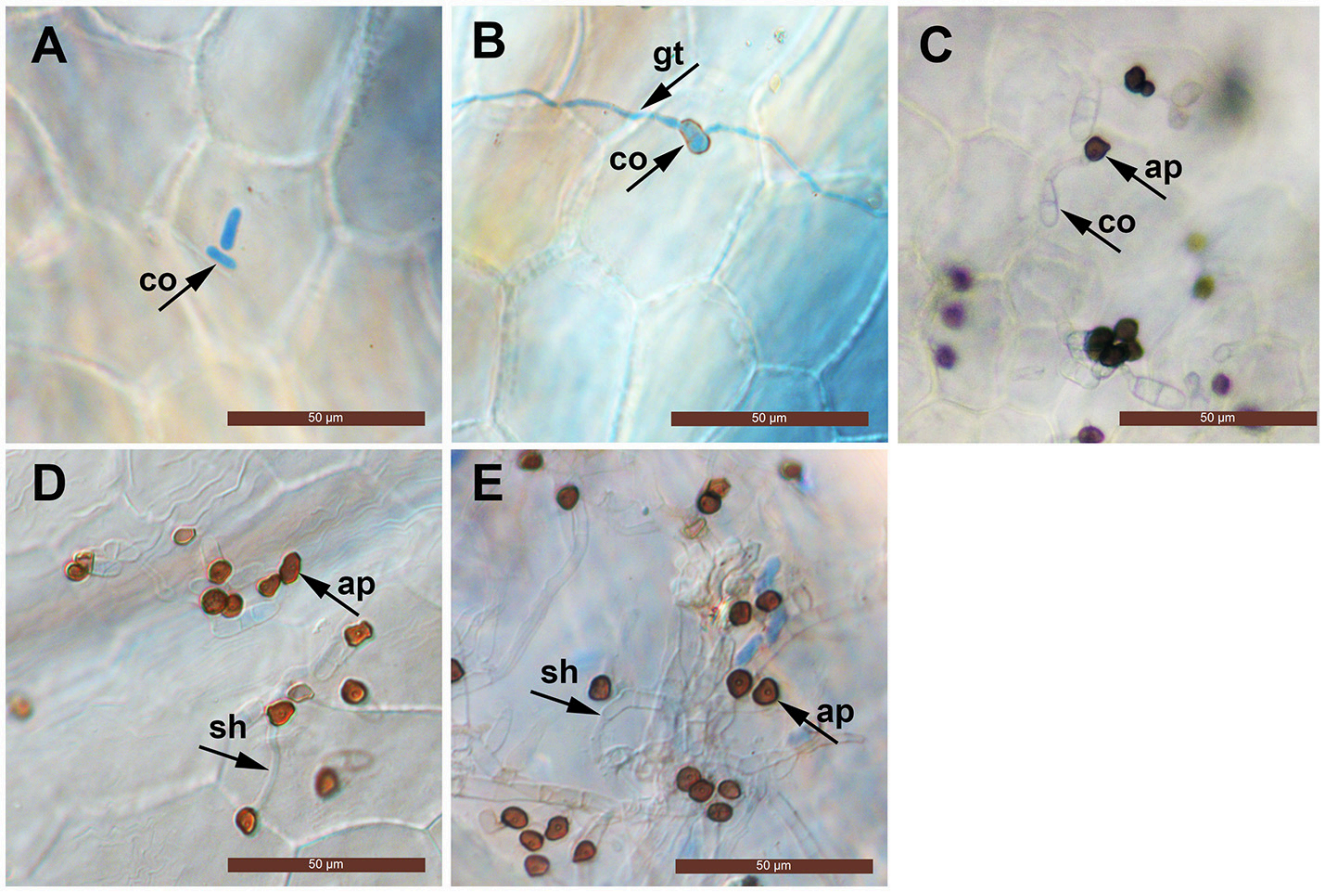
Light micrographs showing infection structures of *C. fructicola* on the leaves of susceptible strawberry cultivar “JiuXiang.” co, conidia; ap, appressoria; gt germ tube. sh, secondary hyphae. Bars = 50 μm. **(A)** Ungerminated conidia at 0 hpi. **(B)** Germination of conidia with germ-tube from one tip at 12 hpi. **(C)** Formation of mature, melanized appressoria 24 hpi. **(D)** Formation of a hyphae which had a morphology that is reminiscent of secondary hyphae (sh) at 72 hpi. **(E)** Development of secondary hyphae (sh) at 96 hpi.

The conidia of *C. fructicola* were oval with a smooth surface at 0 h. By 12 h post inoculation (hpi), conidia geminated and generated a germ tube from both tips. By 24 hpi, mature, melanized appressoria could be found. By 72 hpi, the hyphae had a morphology reminiscent of secondary hyphae in other *Colletotrichum* spp. was observed. By 96 hpi, secondary hyphae were predominant and the leaves developed visible anthracnose symptoms (Figure 1).

### Interactive transcriptomic analyses

For simultaneous analysis of host plant strawberry and its pathogen *C. fructicola*, RNA was isolated from mycelium grown *in vitro* (pathogen control), mock inoculation (strawberry control) or inoculated strawberry leaves at three different time points (24, 72, and 96 hpi). Three biological replicates were pooled and sequenced. We obtained 5.37–14.01 Gb high quality reads from each treatment and aligned them against the reference genomes. The high quality reads were separately aligned to the *C. fructicola* Nara gc5 genome (Gan et al., 2013) and the diploid strawberry progenitor *Fragaria vesca* accession Hawaii 4 genome (Shulaev et al., 2011). For mycelium samples (control of pathogen), 83.77% of reads could be mapped to the *C. fructicola* genome; meanwhile, for samples from infected leaves, the percentage of reads mapped ranged from 0.03% (at 24 hpi) to 0.15% (at 96 hpi) (Table 1). For mock inoculated strawberry leaves, 62.62% of reads could be mapped to the *F*. *vesca* genome; meanwhile, the percentage of reads mapped from samples of infected leaves were61.29% (24 hpi), 60.04% (72 hpi) and 60.12% (96 hpi).

**Table 1:** RNAseq read counts and percentage mapping statistics to *C*. *fructicola* and strawberry genome.

	**Samples**	**Clean reads**	**Genome**	**Total mapped reads**	**Total unmapped reads**	**Unique_match**
Control	JiuXiang (host)	98573510 (100%)	*Fragaria*_*vesa*	61726447 (62.62%)	36847063 (37.38%)	54204716 (54.99%)
	CGMCC3.17371 (pathogen)	42264966 (100%)	Naragc5	35405362 (83.77%)	68596041 (16.23%)	21244745 (50.27%)
Infected mixed transcriptome	24 hpi	88846458 (100%)	*Fragariavesa*	544520336 (61.29%)	34394425 (38.71%)	47606785 (53.58%)
			Naragc5	28221 (0.039%)	88818237 (99.97%)	21726 (0.02%)
	72 hpi	953905321 (100%)	*Fragariavesa*	57270501 (60.04%)	38120031 (39.96%)	48761603 (51.12%)
			Naragc5	53153 (0.06%)	95337379 (99.94%)	45235 (0.05%)
	96 hpi	109226054 (100%)	*Fragariavesa*	65830542 (60.12%)	39104592 (39.93%)	49520288 (50.56%)
			Naragc5	114160 (0.15%)	97823884 (99.88%)	107802 (0.11%)

**Figure 2:**
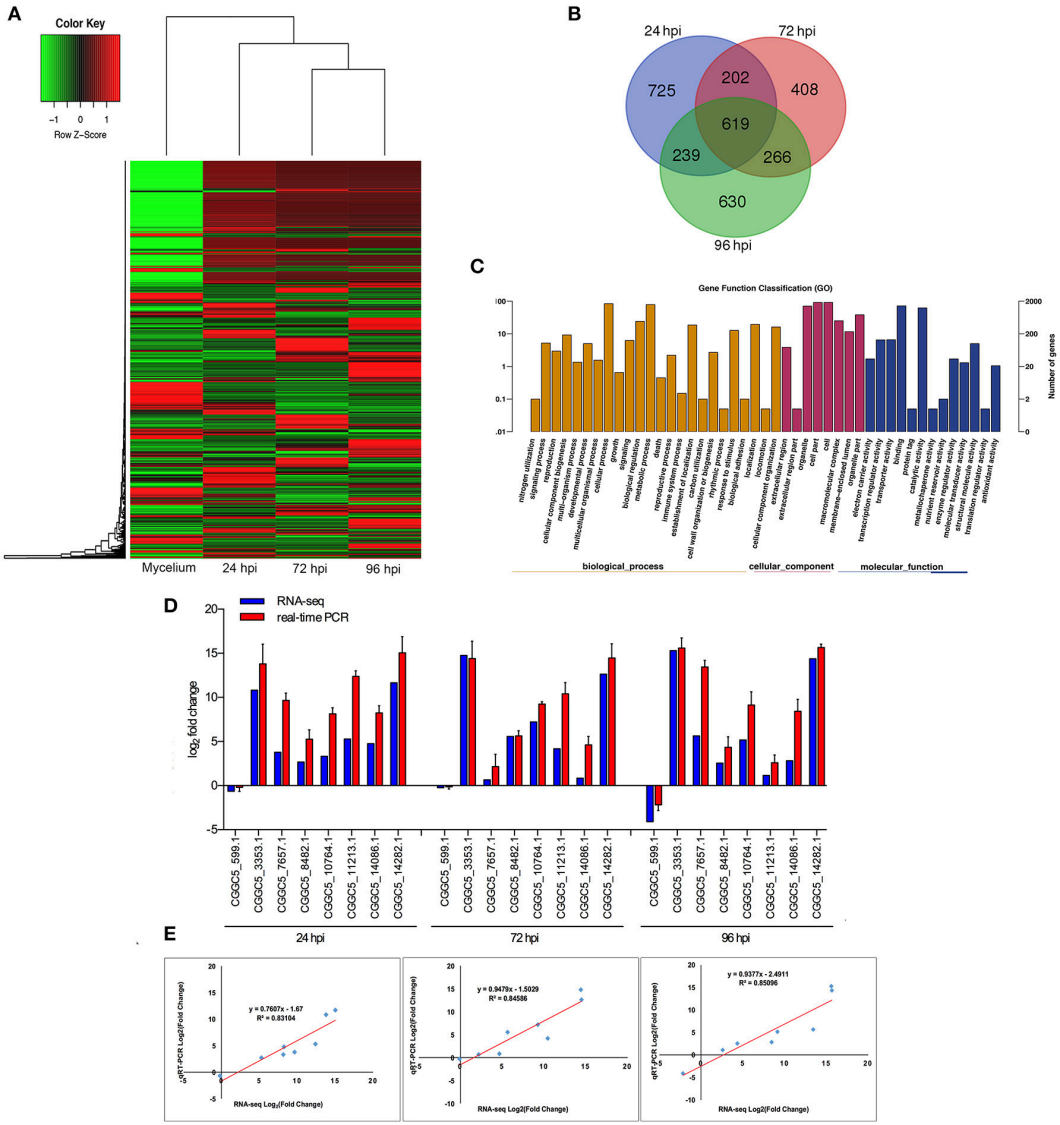
Global aspects of the fungal transcriptome during three stages of fungal development. **(A)** Heatmap of 7811 *C. fructicola* genes that showed at least a 2-fold expression differences with a *p*-value < 0.05. A colored bar indicating the standardized log_2_ (reads per kilobase per million) (RPKM) accompanies the expression profile, ranging from green (−1) to red (1). Hierarchical clustering shows the gene expression clusters. **(B)** Overlap of up-regulated *C. fructicola* genes at each infection stage compared with mycelium grown in PDA medium: 24 hpi (purple), 72 hpi (red), and 96 hpi (green). **(C)** Gene ontology classification of *C. fructicola* transcriptome. The three main GO categories include biological process (yellow), cellular component (red), and molecular function (blue). The x-axis represents the GO annotation categories and the y-axis represents the percentage of total matched genes from a specific category. **(D)** Quantitative analysis of DEGs from *C. fructicola* transcriptome. Comparison of RNA-Seq and qRT-PCR validation results. Eight *C. fructicola* genes were selected for differential expression (DE) confirmations in the same RNA samples used for RNA-seq. Error bars represent the standard error of the mean. The x-axis shows genes in three time points validated in this study; The y-axis shows the log_2_ ratio of *C. fructicola* gene expression in infected strawberry (24, 72 and 96 hpi) vs. mycelium (control of pathogen). **(E)** Correlation between RNA-seq and qRT-PCR data of eight selected *C. fructicola* genes. Expression values were obtained using three techniques replicates and are presented as fold change Log2.

When comparing inoculated strawberry leaves with mycelium grown *in vitro* (pathogen control), there were 7811 differentially expressed genes (DEGs) in *C. fructicola* (2-fold change) (Table S2) (Figure 2A). Venn diagrams of *C. fructicola* up-regulated genes at each fungal colonization stage compared with the mycelium samples revealed an overlap, but also showed distinct stage-specific expression (Figure 2B). In total, 725, 408, and 630 genes were exclusively expressed at 24, 72, and 96 hpi, respectively. We further examined the functional roles of all the DEGs based on 45 categories belonging to three groups using gene ontology (GO) enrichment analysis (Figure 2C). The “catalytic activity,” binding, “transporter activity,” and “transcription regulator activity” categories were the most enriched GO terms in the “molecular function” group. The “metabolic process,” “cellular process,” and “biological regulation” categories were the most enriched GO terms in the “biological process” group. The “cell,” “organelle,” and “cell part” categories were the most enriched GO terms in the “cell component” group (Figure 2C). To validate gene expression data obtained through differential expression analysis, eight candidate effector genes were tested using qRT-PCR. *Actin* and α*-tubulin* were used as reference genes. All eight of the selected genes showed trends similar to those found in the RNA-seq data. The qPCR results were highly consistent with RNA-Seq results, with pearson correlation coefficients ranging from 0.83 to 0.85. These results suggest that the transcriptome analysis was reliable (Figures 2D,E).

**Figure 3:**
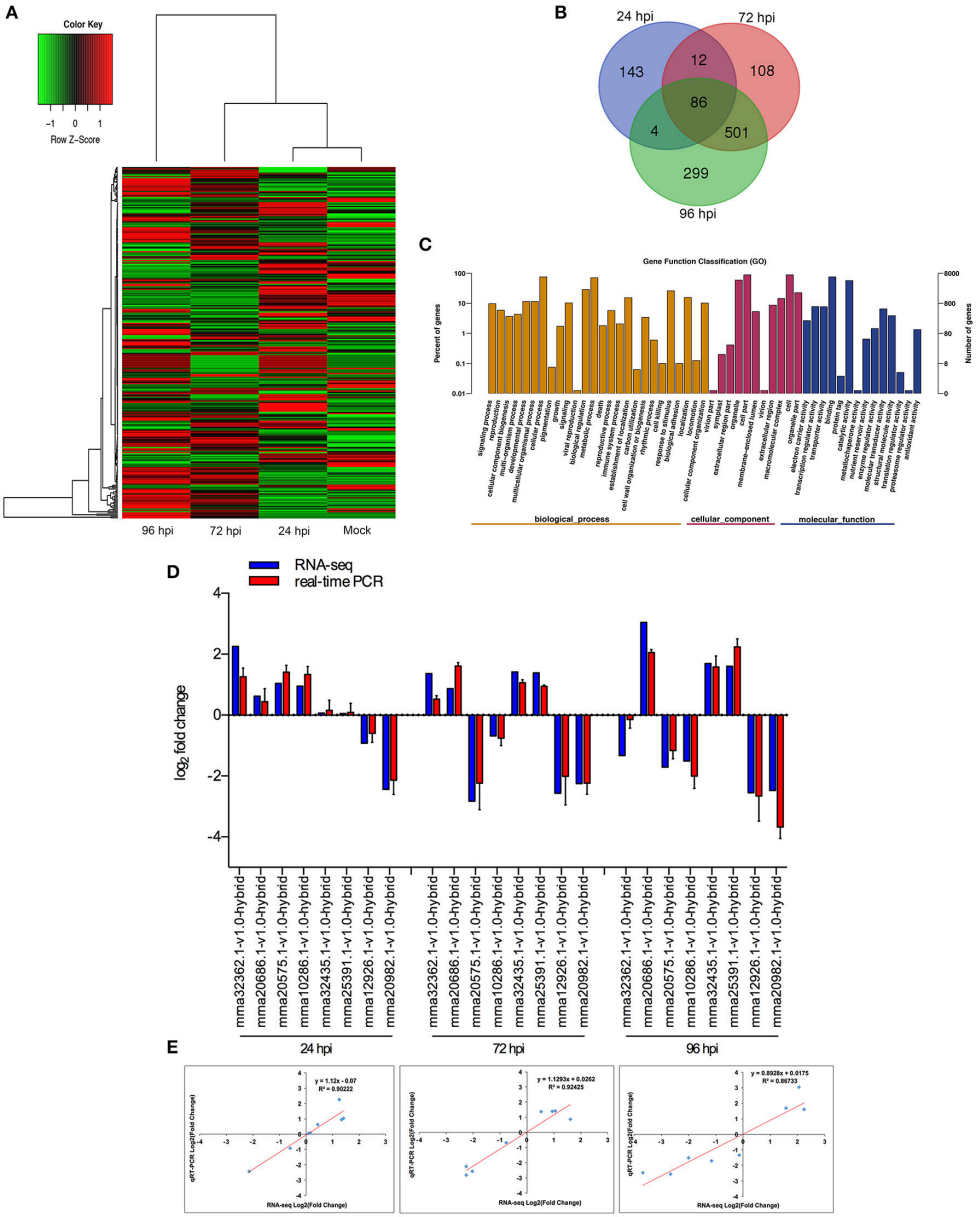
Global aspects of the strawberry transcriptome during three stages of fungal development. **(A)** Heatmap of 2273 strawberry genes that showed at least 2-fold expression difference with *p*-value < 0.05. A colored bar indicating the standardized log_2_ (reads per kilobase per million) (RPKM) accompanies the expression profile, ranging from green (−1) to red (1). Hierarchical clustering shows gene expression clusters. **(B)** Overlap of up-regulated genes in response to each stage compared with mock treatment with water containing Tween−20: 24 hpi (purple), 72 hpi (red), and 96 hpi (green). **(C)** Gene ontology classification of strawberry transcriptome. The three main GO categories include biological process (yellow), cellular component (red), and molecular function (blue). The x-axis represents the GO annotation categories and the y-axis represents the percentage of total matched genes from a specific category. **(D)** Quantitative analysis of DEGs from strawberry transcriptome. Comparison of RNA-Seq and qRT-PCR validation results. Eight strawberry genes were selected for DE confirmations in the same RNA samples used for RNA-seq. Error bars represent the standard error of the mean. *x*-axis shows genes in three time points validated in this study; *y*-axis shows the log_2_ ratio of strawberry gene expression in infected strawberry (24, 72, and 96 hpi) vs. mock inoculated strawberry (control of strawberry). **(E)** Correlation between RNA-seq and qRT-PCR data to eight selected strawberry genes. Expression values were obtained using three techniques replicates and are presented as fold change Log2.

When comparing inoculated strawberry leaves vs. mock inoculation, 2273 DEGs were found in the strawberry (2-fold change) (Figure 3A, Table S14). Venn diagrams of strawberry genes up-regulated in response to each fungal colonization stage were compared with the non-inoculated mock treatment (Figure 3B): 245 genes were up-regulated at 24 hpi. By comparison, 707 and 890 genes were up-regulated at 72 and 96 hpi, respectively; 587 genes were up-regulated at both 72 and 96 hpi, indicating an overlap between those two time points. The DEGs annotated to the GO database were distributed among 47 functional types, including “growth,” “cell,” “response to stimulus,” “metabolic process,” and “signaling” (Figure 3C) (Ke et al., 2014). To validate the gene expression data obtained through differential expression analysis, eight plant hormones and cell apoptosis–related genes were tested for expression profiling using qPCR. *GADPH* were used as the reference gene. The trends in expression levels of the eight selected genes were similar to those found in the RNA-seq data. The qPCR results were highly consistent with RNA-Seq results, with Pearson correlation coefficients ranging from 0.86 to 0.92. The results suggest that the transcriptome analysis was reliable (Figures 3D,E).

### *C. fructicola* transcriptomics

#### Adhesion to the host surfaceand remodeling of cell walls

Fungal hydrophobins are small, secreted, hydrophobic proteins that may be involved in the adhesion of spores to the leaf surface (Tucker and Talbot, 2001). In *Magnaporthegrisea*, the class I hydrophobin MPG1 and the class II hydrophobin MHP1 are both required for full pathogenicity (Talbot et al., 1993, 1996; Kim et al., 2005). In our data, a gene encoding MPG1 (CGGC5_8072) was expressed at high levels throughout the entire course of infection (Table S3). Another secreted fungal protein that binds to hydrophobic surfaces is encoded by HsbA in *Aspergillus oryzae*. This secreted protein binds to artificial polybutylene succinate-co-adipate (PBSA) hydrophobic surfaces and has been shown to recruit a polyesterase, which degrades PBSA and enables the fungus to use it as a carbon source (Ohtaki et al., 2006). Based on the occurrence of the HsbAPfam motif (PF12296), nine homologs of HsbA were discovered in the transcriptome of *C. fructicola*. Analysisof the transcriptomic data (Table S3) suggests that eight HsbA-encoding genes are up-regulated at 24 hpi and that the ninth gene is up-regulated only at a later stage (72 hpi). These data suggest that HsbA-like protein-mediated adhesion likely plays a role during *C. fructicola* appressorium development.

In the first step of invasion, pathogens need plant cell wall–degrading enzymes (PCWDEs) to degrade the plant cell walls and facilitate penetration and migration; meanwhile, remodeling of the pathogen's cell wall is necessary for evasion of PTI (Bellincampi et al., 2014; Oliveira-Garcia and Valent, 2015). The most prevalent PCWDEs during the early stages of *C. fructicola* infection (24 hpi) are pectin-degrading enzymes, such as carbohydrate esterase (CE) family 1, glycoside hydrolase (GH) family GH3, GH31, and several pectate lyases (PL1, PL3) (Table S4). Pectin is the main component of plant cell walls. The depolymerization of pectin makes the plant cell wall more vulnerable and accessible to other PCWDEs (Malinovsky et al., 2014). An increase in the number and diversity of the GH families that facilitate plant cell wall breakdown was observed at 96 hpi, including GH28 (polygalacturonase), GH31 (α-glucosidase), GH47 (α-mannosidase), and GH105 (rhamnogalacturonylhydrolase), as well as pathogen wall modification, such as GH-3 (β-1,3-glucan) and GH-18 (chitinase) (Figure S1, Table S4).

The transcription of an abundant class of genes encoding enzyme inhibitors was increased, including glucanase inhibitor proteins that bind to and inhibit host endo-β-1,3-glucanases of glycosyl hydrolase (GH) family 16; this was especially prevalent during 72 hpi. These proteins may impede the degradation of the fungal cell wall and prevent it from being recognized by the plant (Esquerré-Tugayé et al., 2000). Notably, three genes containing the carbohydrate-binding module 50 (CBM50), which binds to chitin (LysM domain-containing proteins), were up-regulated at 72 and 96 hpi (Table S4). The binding of chitin by pathogen proteins could help it to avoid recognition by host chitin receptors and suppress chitin-induced host immunity (de Jonge and Thomma, 2009).

#### Primary metabolism: fatty acid oxidation and the glyoxylate cycle were activated during the early stage of *C. fructicola* infection

During the infection process, pathogenic fungi usually encounter nutrient deprivation in the host before gaining access to sufficient nutrients to successfully colonize living tissue (Lee et al., 2009). We observed significant up-regulation of the key genes involved in two primary metabolic pathways (fatty acid oxidation and the glyoxylate cycle).

**Figure 4:**
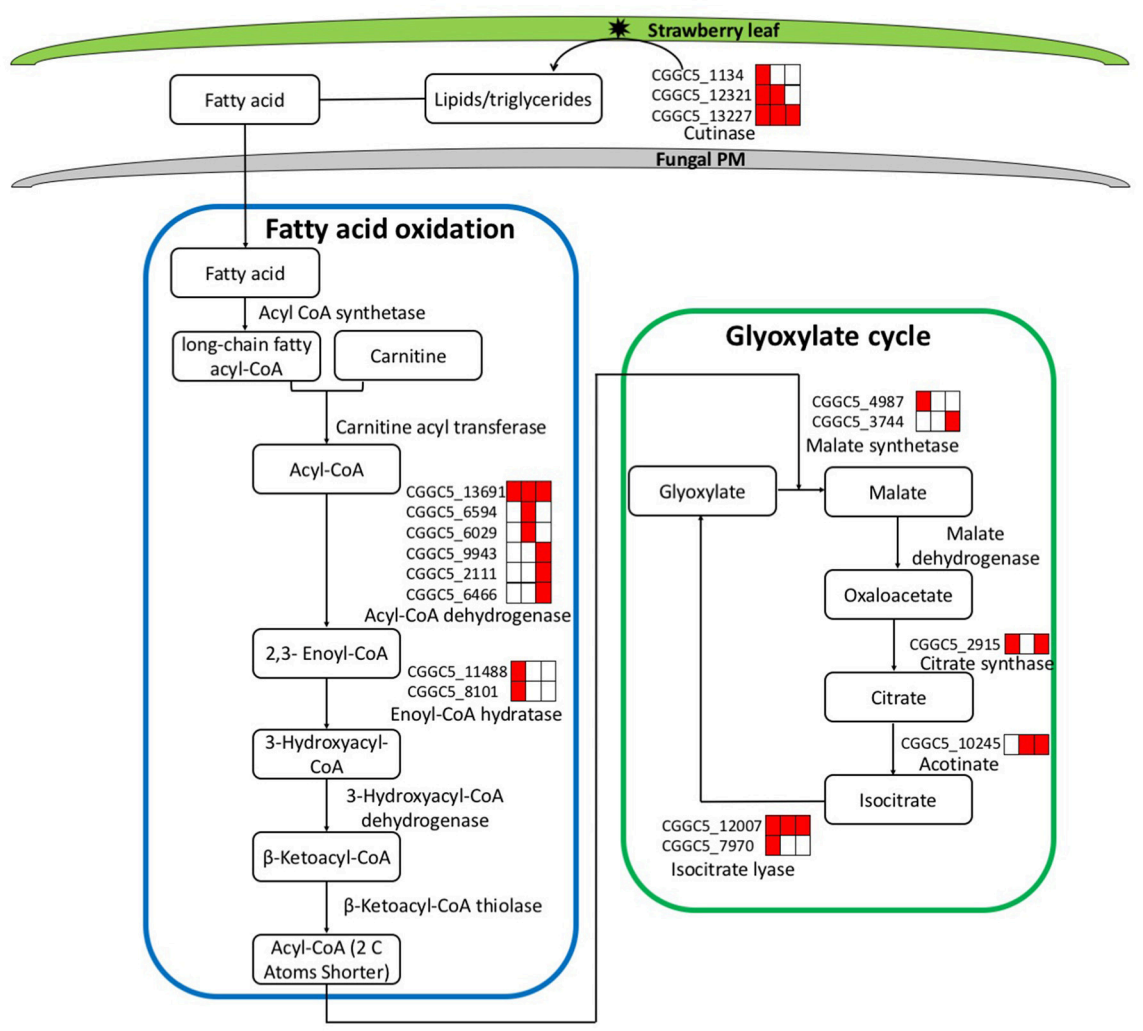
Leaf infection induces the expression of *C. fructicola* genes involved in the beta-oxidation of lipids and fatty acids and the up-regulation of the glyoxylate pathway. Red coloring indicates up-regulation and no shading represents no significant change relative to the expression levels *in vitro* culture (adjusted *p* < 0.05). FA, Fatty acids; PM, plasma membrane.

During the first step of infection, fungi produce extracellular degrading enzymes. We observed significant up-regulation of cutinase–coding genes during *C. fructicola* infection (Table S4). This process provides the materials needed for fungal fatty acid oxidation. Thereafter, fatty acid synthesis and subsequent transport were bothactivated at 24 and 72 hpi, respectively, and were characterized by the up-regulation of the genes encoding Acyl-CoA dehydrogenase and Enoyl-CoA hydratase. Acyl-CoA is the basic currency of carbon metabolism within the cell (Strijbis and Distel, 2010). In our study, two genes encoding key enzymes of the glyoxylate cycle (citrate synthase and isocitratelyase) were highly induced at 24 hpi. This indicates that the use of Acyl-CoA, which is derived from fatty acid β-oxidation, may provide carbon skeletons for anabolic processes via the glyoxylate cycle early in infection (Figure 4, Table S5).

The expression patterns demonstrate that *in vitro*, where abundant nutrition sources are available in the culture medium, *C. fructicola* tends to rely on glycolysis and the tricarboxylic acid cycle for energy production. By contrast, during the early stages of infection where there are limited sources of nutrition, *C. fructicola* tends to rely on the mobilization of stored lipids through fatty acid oxidation to form Acyl-CoA, which is further assimilated via the glyoxylate cycle to supply energyto cells (Table S6).

#### Secondary metabolism: melanin biosynthesis is a pivotal process during appressorium development

Secondary metabolites produced by *Colletotrichum* are known to contribute to pathogenicity (Gan et al., 2013). There were 11 secondary metabolite backbone genes up-regulated in our transcriptomic data (Table S7). Three polyketide synthases (PKSs)-encoding genes (CGGC5_9803, CGGC5_12144, and CGGC5_14485) were significantly up-regulated at 24 hpi. CGGC5_14485 was homologous with ALB1, which encodes a PKS that catalyze the first step in the melanin biosynthesis of *M*. *grisea* (Chumley, 1990). The other five secondary metabolite backbone-forming genes were highly up-regulated at 96 hpi; this coincides with the necrotrophic stage of *C. fructicola* (Table S7).

Secondary metabolites associated with sporulation can be placed into three broad categories, including pigment. Melanin, a pigment located in the cell wall of appressoria, can provide mechanical strength that aids in tissue penetration (Eisenman and Casadevall, 2012). Many fungi synthesize melanin via the dihydroxynaphthalene (DHN) pathway. DHN is produced from 1,3,6,8-tetrahydroxynaphthalene (1,3,6,8-THN) via a sequence of reduction and dehydration reactions with the intermediates scytalone,1,3,8-trihydroxynaphthalene and vermelone (Eisenman and Casadevall, 2012). The expression profiles of *C. fructicola* genes encoding enzymes of the DHN melanin biosynthesis pathway were all up-regulated at 24 hpi (Table S8). Melanin biosynthesis is a pivotal process during appressorium development and seems to play a role in *C. fructicola* pathogenesis.

#### Transporters and detoxification of compounds protect *C. fructicola* from host defense

Plant pathogens must protect themselves, especially during infection when they are likely to encounter host defense mechanisms (Göhre and Robatzek, 2008). One way fungi accomplish this task while overcoming intercellular toxin accumulation is via efflux pumps, in particular ATP-binding cassette (ABC) transporters and transporters of the major facilitator superfamily (MFS) (Coleman and Mylonakis, 2009). In our data, several ABC-transporter-encoding genes were up-regulated at all three time points post infection (Table S9). The ABC transporters in other fungi have shown to play an important role in fungal resistance. For example, BcatrB from *Botrytis cinerea* (homolog of CGGC5_15191) and NhABC1 from *Fusariumsolani f*. *sp*. *pisi* (homolog of CGGC5_9023) were shown to be responsible for the efflux of plant-derived defense compounds (Stefanato et al., 2009; Coleman et al., 2011). A search for genes with the Pfam membrane transporter motifs PF00083 (a signature of saccharide) and PF07690 (MFS signature) identified 83 up-regulated genes (Table S10). Of these, 56 were up-regulated at 24 hpi. Genes encoding sugar and MFS transporters were also highly up-regulated; (Figure S2A). In *Cercospora nicotianae*, a MFS transporter gene *CTB4* (homologs of CGGC5_9801), encodes a cercosporin efflux pump that contributes to self-protection from cercosporin, subsequently facilitating the virulence of these pathogens on their host plants (Choquer et al., 2007). The significant up-regulated ABC and MFS transporter genes at 24 hpi may suggest that these transporters play a critical role in protection against plant defense compounds during the early stage of infection (Del Sorbo et al., 2000).

Cytochrome P450s (CYP450) are involved in detoxification and contribute to host interactions (Nelson, 1999). We observed 36 genes encoding CYP450 (PF00067) that were simultaneously up-regulated at 72 hpi. A recent publication showed that P450s were specifically involved in virulence as well as both asexual and sexual development. Most importantly, P450s seem to play redundant roles in the degradation of xenobiotics in *Fusarium graminearum*; *CYP51* genes in *F*. *graminearum* (homologs of CGGC5_10696, CGGC5_2390) mediate differential sensitivity to sterol demethylation inhibitors (Shin et al., 2017). CYP450 contributes to the production of mycotoxins and the detoxification of host metabolites in other fungi (Crešnar and Petric, 2011). The up-regulation of several P450 genes in our data may suggest a special role for theCYP450-mediated detoxification produced by the plant at this stage (Table S11, Figure S2B).

#### Defining *C. fructicola* candidate effectors

Fungal effectors are secreted molecules that modulate the interaction between the fungus and its host (Lo Presti et al., 2015). There are 2042 genes (13.2%) that encode secreted proteins in the genome of Nara gc5 (Gan et al., 2013). The current transcriptomic data show that some homologs of known effectors from other phytopathogens were differentially expressed after *C. fructicola* infection. For example, the gene encoding CGGC5_2199 (homolog of Cmu1) was up-regulated at 24 hpi. The effector Cmu1 counteracts salicylic acid (SA)-dependent immunity in the host, functioning as a chorismate mutase to reduce the levels of the SA precursor chorismate (Djamei et al., 2011). The genes encoding CGGC5_10914 (a homolog of Ecp6) were up-regulated at 96 hpi; this is consistent with the previously reported expression patterns of this gene. Previous reports showed that the expression of Ecp6 gene was gradually up-regulated and maximal at 13 dpi during the interaction of *Cladosporium fulvum* with tomato (Bolton et al., 2008). Ecp6 has identified to be a novel virulence factor through sequestering chitin oligosaccharides to prevent elicitation of plant immunity (de Jonge et al., 2010). The genes encoding for CGGC5_10914 (a homolog of CHEC30) were up-regulated at 72 and 96 hpi. *C*. *higginsianum* effector CHEC30 was appeared to be preferentially expressed at biotrophic stage (Kleemann et al., 2012). The expression patterns of other homologs of known effectors are listed in Table S12.

**Figure 5:**
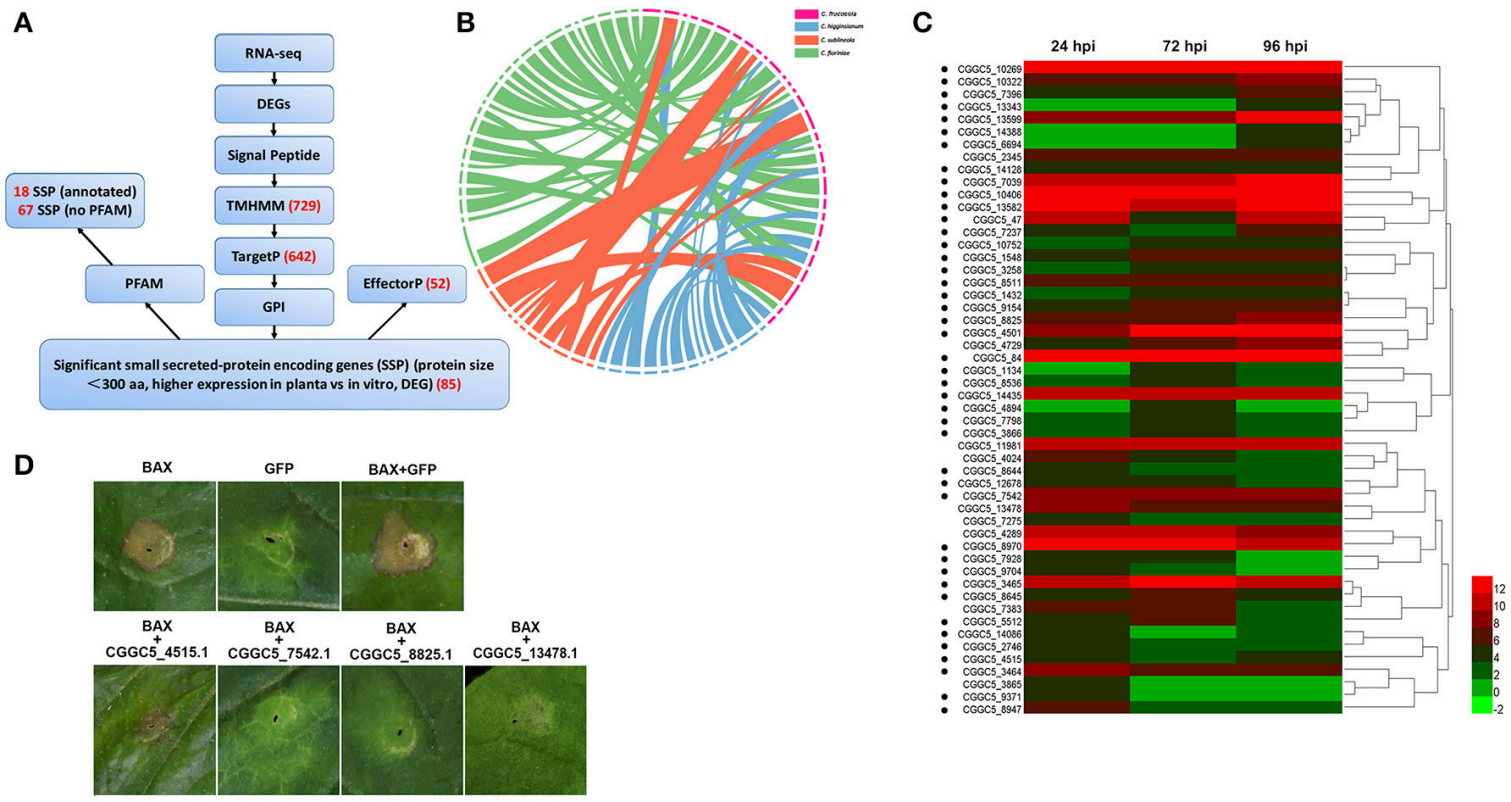
*C*. *fructicola* secretome prediction pipeline. **(A)** Features used to predict and classify effectors of *C*. *fructicola*. Secreted proteins were predicted using SignalP, TMHMM, TargetP, GPI and EffectorP. Pfam was used to verify the biological functions enriched in the secretome of *C*. *fructicola*. The number of selected proteins is indicated in red font. DEG, differentially expressed gene; RNA-seq, RNA-sequencing. **(B)** Comparative analysis of *C*. *fructicola*-secreted proteins with other *Colletotrichum spp*: *C*. *fructicola* (red), *C*. *higginsianum* (blue), *C*. *sublineola* (orange), and *C. fioriniae* (green). We chose the 642 predicted secreted proteins to draw a Circos plot. The Circos plot shows secreted proteins as ideograms. A stringent *E*-value (1e-50) was used to identify potential homologs and only the top BLAST hit was included in the Circos plot. Ribbon links convey the linked region between two ideograms with over 90% amino acid residue identities. **(C)** Heat map of the 52 predicted small secreted protein (SSP)-encoding genes differentially expressed during infection at different time points (24, 72, and 96 hpi). Hierarchical clustering shows gene expression clusters. The expression of each gene is based on regularized logarithmic transformation of the average of the best three biological replicates using DESeq2. The color bars represent the values of log_2_fold change, ranging from green (−2) to red (12). Candidate effector proteins predicted to be N/O-glycosylated are indicated with black dots. **(D)** Suppression of Bax-induced cell death by *C. fructicola* candidate effectors in *Nicotiana benthamiana* (*Nb*) leaves. *Agrobacterium tumefaciens* (*Ag*) strains expressing individual *C. fructicola* SSPs and Bax were infiltrated into *Nb* leaves. *Ag* carrying GFP was used as a negative control, and Bax was used as a positive control. Bax-induced cell death was scored at 3–5 days after infiltration.

In addition to these known effectors, candidate effectors were identified in the transcriptome of *C. fructicola*via a pipeline (Figure 5A) that shares common features with those described for filamentous plant pathogens (Petersen et al., 2011; Guyon et al., 2014). We first refined the prediction by combining the predictions from SignalP4.1 and TMHMM to identify 729 proteins that had secretion signals but did not have transmembrane helices (Petersen et al., 2011). Next, GPIsom removed 87 proteins harboring glycophosphatidyl inositol anchor motifs, which likely represent surface proteins rather than secreted effectors (Fankhauser and Mäser, 2005). This resulted in 642 predicted secreted proteins (SPs). A Circos plot was generated to visualize the peptide sequence similarities between these SPs and their homologs. These homologs show a 90% peptide sequence identity, which indicates that these SPs have striking similarities tothose of other *Colletotrichum* species (Figure 5B). In total, 294 of the 642 SPs were small (<300 aminoacid) secreted proteins (SSPs). We observed 85 SSPs that were highly expressed during infection. These 85 SSPs were evaluated with the EffectorP prediction tool, which is usable for both apoplastic and cytoplasmic effectors (Sperschneider et al., 2016). 52 SSPs were ultimately identified as the most likely set of *C. fructicola* effectors (Figure 5C). Post-translational modification of effectors influences their function. Glycosylation likely impacts effector function by modifying protein stability, as well as conformation or resistance to host proteases (Doehlemann et al., 2014). Here, 90.6% of candidate effectors carried at least one predicted N/O-glycosylation site (Figure 5C, Table S13).

To identify the function of the candidate effectors, four highly expressed SSPs were chosen for a transient assay in *Nicotiana benthamiana*. The signal peptides of these candidate effectors were first removed to enable the genes to be expressed stably in plant cells. The four candidate effectors that partially or fully suppressed Bax-mediated programmed cell death in tobacco leaves (Figure 5D).

### Strawberry transcriptomics

#### Cell wall fortification

Improving cell wall lignin content is one of the common plant defense mechanisms. In our study, inoculation induced the expression of strawberry 4-coumarate-CoA ligase and disease resistance-responsive (dirigent-like) proteins. These proteins are involved in lignin biosynthesis (Lee et al., 1995; Davin et al., 1997). Several dirigent-like protein-encoding genes were up-regulated during the three stages of infection (Table S15).

A number of cell wall synthesis genes were also down-regulated during the course of *C. fructicola* infection. For example, six of the 10 cellulose-synthase-like genes, including CSLC5 and CSLD3 homologs (Dai et al., 2011; Almeida et al., 2015), were down-regulated at 72 and 96hpi (Table S15). Similar changes (i.e., replacement of cell wall components) have been observed in *Arabidopsis* CESA3-deficient mutants (Caño-Delgado et al., 2003). Reduced cellulose synthesis has been suggested to invoke a wide range of cellular responses, including activating lignin synthesis and defense responses mediated via JA and ethylene (ET) signaling pathways (Caño-Delgado et al., 2003; Hamann, 2012).

#### Pathogen perception: activation of PAMP-triggered immunity (PTI) and effector-triggered immunity (ETI)

Through evolution, plants have developed a two-step innate immunity system to prevent pathogen invasion. The first step involves the recognition of pathogen-associated molecular patterns (PAMPs) through plant pattern recognition receptors (PRRs); this subsequently triggers the PTI (Nicaise et al., 2009). Plant PRRs are either surface-localized receptor-like kinases (RLKs) or receptor-like proteins (RLPs), which usually contain ligand-recognition domains, such asleucine rich repeats (LRRs) and lysine motifs (LysMs). Chitin is the major constituent of fungal cell walls and serves as a classic fungal PAMP that can be recognized by several typical plantPRRs (e.g., chitin elicitor receptor kinase 1 [CERK1], chitin elicitor-binding protein [CEBiP]) (Kaku et al., 2006; Miya et al., 2007). We did not observe significant up-regulation of genes encoding CERK1 or CEBiP in *C. fructicola–*infected plants. However, a set of strawberry RLKs were differentially expressed during infection. 53% (23/43) and 60% (26/43) of RLKs were significantly up-regulated at 72 and 96 hpi, while 23% (10/43) of RLKs were down-regulated at both 72 and 96 hpi. (Table S16).

ETI involves the direct or indirect recognition of effectors by plant resistance (R) proteins (Chisholm et al., 2006). The execution of ETI is mediated by intracellular immune receptors (e.g., NBS-LRR proteins) in order to perceive secreted virulence effectors (Zipfel, 2014). In our study, five CC-NBS-LRRs, two TIR-NBS-LRRs, and five NB-ARC domain-containing proteins were differentially expressed (Table S17). Among them, two NB-ARC genes were down-regulated while six were up-regulated during all three stages of infection. However, the successful infection of *C. fructicola* indicated that although some R genes were up-regulated at transcriptional level, they might not fully exert their function and block fungal infection.

#### Core immune phytohormones: SA is effectively triggered

In this study, 50, 88, and 100% of SA-signaling genes were significantly up-regulated at 24, 72, and 96 hpi, respectively. The nonexpressor of pathogenesis-related genes 1 (NPR1) is the key gene that mediates defense gene expression induced by SA (Mou et al., 2003). We observed an up-regulation of *NPR1* at 72 and 96 hpi. The SA pathway activates many downstream defense related genes, including *PR* genes (van Loon et al., 2006). The PR4 family encodes chitinase and can be induced by *F*. *culmorum* infection, SA treatment, or JA treatment in wheat (Bertini et al., 2003). In our study, two homologous PR4 genes, PR4a and PR4b, were up-regulated (by 2.3- and 2.4-fold, respectively) at 24 hpi. By 72 hpi, they were up-regulated by 29- and 38-fold, respectively; this increased further to 49- and 60-fold by 96 hpi (Table S18). In addition, one gene coding the cysteine-rich secretory protein (CRISP) was highly expressed at 96 hpi. The homolog of the CRISP in plants is the PR-1 protein; this protein plays an important role in anti-fungal activities (Niderman et al., 1995). These highly up-regulated PR genes seem to indicate their potential role in *C. fructicola* resistance. However, in our previous reports, several relevant immune-related genes were chosen for qRT-PCR assay to evaluate their gene expression level between susceptible (JiuXiang, JX) and less-susceptible plant (Sweet Charlie, SW) (Zhang et al., 2016). Specifically, several SA-related genes, including PR1a and PR5 were up-regulated in both JX and SW. Notably, the gene expression level in less-susceptible plant were significantly higher than that in susceptible plant. For example, at 96 hpi, PR1a was up-regulated by ~30- and 400-folds in susceptible and resistant plant, respectively. Similarly, PR5 was up-regulated by ~10- and 600-folds in susceptible and resistant plant, respectively. These results suggest that although several SA-related genes seem to be up-regulated in successful infection, the extent of the up-regulation is significantly less than that in resistant infection. The relative low level of these genes may not be able to inhibit fungal infection in susceptible infection.

JA is an important plant hormone which plays a role in regulating plant development and stress responses. The largely antagonistic functions of JA and SA have been suggested for plant response to pathogen infection (Verma et al., 2016). There also exists cooperative interplay between JA and SA during early ETI. In parallel to SA accumulation in Arabidopsis, the endogenous JA level also increases during ETI induction, which may reduce plant susceptibility to necrotrophic pathogens in the neighboring tissue; this elevation of JA is dependent on the degradation of JAZ mediated by direct interaction with NPR3-NPR4 (Liu et al., 2016). Lipoxygenases (LOX), especially LOX2, are required for wound-induced JA accumulation in *Arabidopsis* (Bell et al., 1995). In this study, the gene encoding LOX1 was reduced by 10.5-, 3.6-, and 4.3-fold at 24, 72, and 96 hpi, respectively. In addition, the gene encoding LOX2 was significantly down-regulated at 96 hpi. JA biosynthesis is possibly regulated by post-translational modifications of pre-existing enzymes, such as allene oxide cyclase (AOC) (Schaller and Stintzi, 2009). Similar to LOX, the gene encoding AOC4 was reduced by 3.1-, 3.0-, and 4.2-fold at 24, 72, and 96 hpi, respectively (Table S19). These results indicate that JA signaling maymainly inhibited by *C. fructicola* during the entire infection process.

JA and ET operate synergistically in regulating defense responses after pathogen infection (Verma et al., 2016). ET and the related signaling pathway can positively or negatively regulate plant immunity; additionally, this pathway exhibits extensive cross-talk with the SA and JA pathways (Sato et al., 2010; Shakeel et al., 2013). The gene encoding ethylenereceptor2 was down-regulated at 24 hpi. Ethylene response factors (ERFs) act downstream of the intersection between the ET and JA pathways. These transcription factors, such as ERF1, are key elements regulating defense responsive genes (Lorenzo et al., 2003). In our study, the genes encoding the ethylene responsive element–binding factor 2 (EREBF2) and the ethylene-forming enzyme (EFE) were mildly up-regulated after *C. fructicola* infection. Meanwhile, the other eight genes encoding the ethylene-responsive transcription factor were significantly down-regulated at three time points after infection (Table S20).

**Figure 6:**
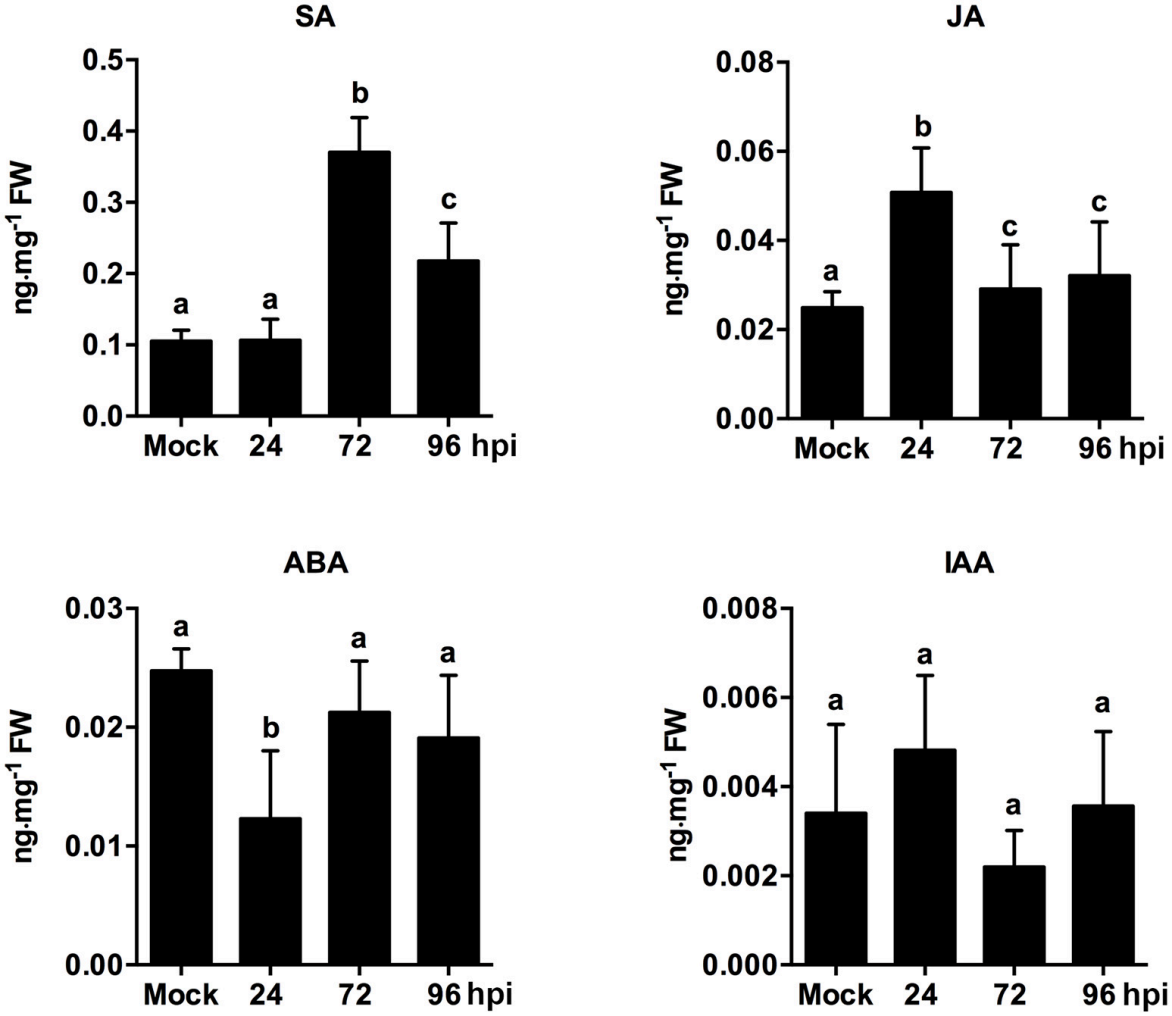
Analysis of phytohormone (SA, JA, ABA, IAA) levels in strawberry infected by *C. fructicola* at 24, 72, and 96 hpi. The y-axises of histogram of phytohotmones represent ng mg^−1^ fresh weight. The error bars represent standard deviations based on two biological replicates. Phytohormone levels followed by the same lowercase letter are not significantly different at *p* < 0.05 using an ANOVA.

The endogenous hormone contents of strawberry leaves during *C. fructicola* infection were analyzed to further confirm the role of plant hormone signaling in fungal infections. The results showed significant up-regulation of SA content at 72 and 96 hpi. Meanwhile, JA content was mildly up-regulated at 24 hpi, but then down-regulated at 72 and 96 hpi (Figure 6). Our results regarding SA and JA signaling were consistent with previous reports, which demonstrated that the primary mode of interaction between SA and JA pathways was mutual antagonism (Doares et al., 1995; Clarke et al., 2000; Gupta et al., 2000). SA is generally involved in the activation of defense responses against biotrophic and hemi-biotrophic pathogens. By contrast, JA and ET are required for resistance against necrotrophic pathogens (Glazebrook, 2005; Bari and Jones, 2009). In our study, the inhibition of ET and JA signaling may be triggered by *C. fructicola* infection. Alternatively, *C. fructicola* induced the activation of SA signaling, which may indirectly inhibit ET and JA signaling.

#### Other hormone signaling

ABA prominently contributes to plant defense response against both abiotic and biotic stress conditions (Fitzpatrick et al., 2011). ABA shows negative interaction with JA-signaling in the modulation of Arabidopsis resistance to the necrotrophic fungi but it can also positively regulate the resistance to some biotrophic pathogens (Denance et al., 2013). Glycosyl hydrolases (GH) belong to a large family of proteins implicated in carbohydrate metabolism andthe remobilization of sugars. ABA significantly increased the activity of α-L-Arabinofuranosidase, one member of glycosyl hydrolase (GH) family, suggesting that some GH might be associated with ABA mediated processes (Alayon-Luaces et al., 2008). Two glycosyl hydrolase superfamily genes and one glycosyl hydrolase family 32 gene were up-regulated at 72 hpi. Meanwhile, five other GH genes were down-regulated at 72 and 96 hpi (Table S21).

Emerging evidence indicates that auxin-signaling genes regulate resistance to different plant pathogens (Denance et al., 2013); additionally, it is possible that components of the auxin synthesis, signaling, and transport pathways could be utilized by pathogens to increase colonization efficiency (Denance et al., 2013). In our study, the temporary up-regulation of auxin related genes, such as auxin-responsive GH3 family protein and indole-3-acetic acid 7 (IAA7), was observed at 24 hpi. However, these auxin-related genes were significantly down-regulated during the middle and late stages of infection. For example, the expression of the early auxin-responsive gene (IAA7) was increased by 7.1-fold at 24 hpi, but was then decreased by 1.6- and 1.8-fold at 72 and 96 hpi, respectively (Table S22). An analysis of phytohormones levels revealed that ABA content was only mildly inhibited at 24 hpi. Other than this, ABA and IAA content were not significantly different at any point during infection (Figure 6). ABA play a negatively role in plant resistance to certain necrotrophic fungi (Audenaert et al., 2002; AbuQamar et al., 2006). A negative effect of auxin signaling on plant resistance to Necrotrophic Fungi was recently described (Llorente et al., 2008). In our present study, since ABA and auxin pathways were not significantly triggered during infection, these two pathways may not play an equally important role as SA, JA, and ET pathways. However, the precise function of these two hormone pathways needs to be further analyzed.

#### Reactive oxygen species production increases accompanied by the obvious pathological changes during the late stage of *C. fructicola* infection

Reactive oxygen species (ROS) in plants are normally generated as by-products of oxidative phosphorylation and other diverse biosynthetic pathways (Almeida et al., 2015). The acute accumulation of ROS leads to an oxidative burst that induces cell death and restricts further pathogen infection (Apel and Hirt, 2004). Therefore, generation of ROS is usually accompanied by visual symptoms (i.e., hypersensitive responses or necrosis) in plants. In our study, there were obvious pathological changes at 96 hpi. Seven peroxidases (PODs) were significantly up-regulated at 96 hpi. Of the seven, two were up-regulated by 52- and 131-fold at 96 hpi. Meanwhile, another was up-regulated by 30- and 52-fold at 72 and 96 hpi, respectively (Table S23). The significant up-regulation of POD-encoding genes at 96 hpi may indicate the activation of plant defense mechanisms.

#### Various transcription factors were involved in the regulation of immune responses during *C. fructicola* infection

During PTI and ETI, plants trigger a diverse array of immune responses, such as hormone signaling, ROS generation, and MAP kinase (MAPK). The signal transduction of these immune responses relies on a regulatory network of transcription factors (TF) (Moore et al., 2011). A subset of TF families, such as WRKY, NAC, bHLH, ERF/AP2, MYB, and bZIP, play a role in the regulation of immune responses. WRKY transcription factors belong to one of the largest families of transcriptional regulators in plants, and are key regulators of PTI and ETI (Eulgem and Somssich, 2007). In this study, we identified a total of 12 differentially expressed WRKY factors (Table S24). Nine genes encoding WRKY factors, including WRKY75, WRKY50, and WRKY23, were significantly up-regulated during the middle-to-late stages of *C. fructicola* infection. Genes encoding three members of the WRKY family, including WRKY33, were down-regulated at all three stages during infection. *Arabidopsis* WRKY33 is a key regulator of host immunity and also regulates the expression of some other WRKY factors as well as defense-associated genes (Birkenbihl et al., 2012). Interestingly, a strawberry homolog of the upstream regulatory gene, mitogen-activated protein kinase 3 (MPK3), was also down-regulated at all stages during infection.

In addition to the WRKY family, the NAC family of proteins is another group of transcriptional regulators that have been identified as important immune components (Jensen et al., 2008). Our study identified six NAC domain-containing proteinsthat were mainly induced at the middle-to-late stages of infection (Table S25). Like WRKY and NAC, other TFs, such as bHLH and ERF, were also differentially expressed after *C. fructicola* infection (Tables S20, S26). The differential expressions of these TFs as observed in the present study suggest that they may play a potential role in the interaction between *C. fructicola* and strawberry.

#### Fungal–plant interactions

**Figure 7:**
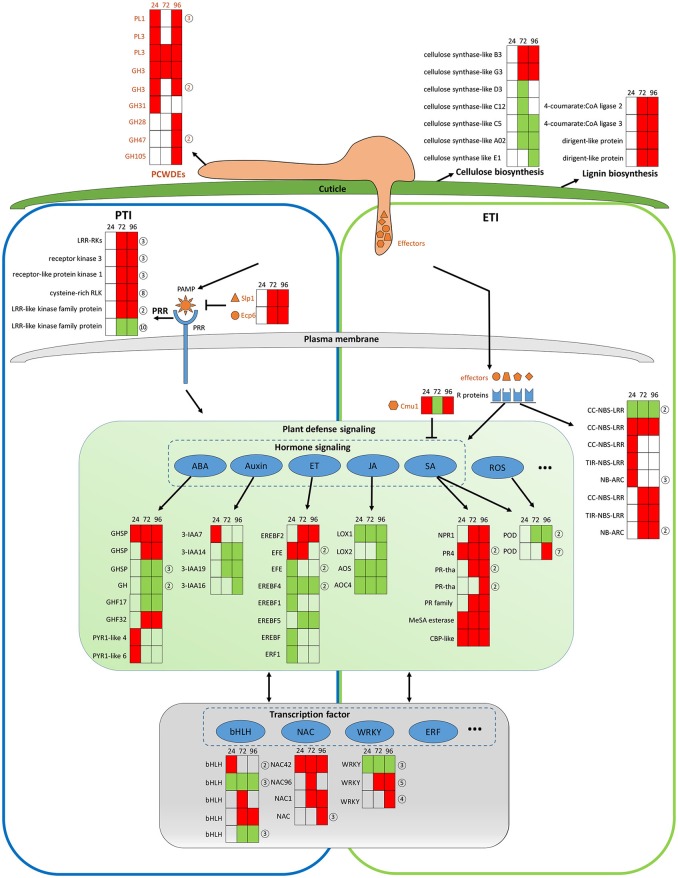
General model of *C. fructicola*–strawberry interaction. The first step of infection includes the fortification of cell wall by strawberry vs. the degradation of the plant cell wall by *C. fructicola* (upper panel). After *C. fructicola* invasion, fungus PAMPs are sensed by host PRRs (PTI panel, left). Fungus secret numerous effectors into and around host cells by invasive hyphae to inhibit plant defense responses (such as PTI) or facilitate colonization. As a response, R proteins recognize effectors and initiates ETI (ETI panel, right). Plant subsequently initiates downstream anti-fungal innate immunity such as SA, ROS signaling (green panel). Various TFs, such as WRKY and NAC, are involved during the whole process of *C. fructicola* infection (gray panel). Red coloring indicates up-regulation, green coloring indicates down-regulation, and no shading represents no significant change (infected leaves at 24, 72, and 96 hpi vs. mock treatment with water containing Tween −20) (adjusted *p* < 0.05). The orange shapes represent the effectors elicited by the fungus. The blue shapes represent R proteins. The full name and gene accession numbers of differentially regulate genes are listed in Tables S4, S12, S15–S26. The numbers in the circles behind the heat maps represent the numbers of the differentially regulated genes in the indicated family. Specifically, PLs and GHs are listed in Table S4. LRR-RLKs, receptor kinase 3, receptor-like protein kinase 1, and cysteine-rich RLK-encoding genes are listed in Table S16. CC-NBS-LRR-and NB-ARC-encoding genes are listed in Table S17. GHSP-and GH-encoding genes are listed in Table S21. EFE-and EREBF5-encoding genes are listed in Table S20. PR4-and PR- encoding genes are listed in Table S18. The POD-encoding gene is listed in Table S23. The bHLH-encoding gene is listed in Table S26. The WRKY-encoding gene is listed in Table S24.

The transcriptomic data give a picture of how adapted *C. fructicola* invades host cells and completes the infection process as strawberry works to mitigate immune suppression and slow the infection rate, albeit at a serious price. It should be noted that *C. fructicola* ultimately, successfully infects the plant and causes symptoms; this means that *C. fructicola* wins the “battle” against host resistance. Our transcriptomic data demonstrate that *C. fructicola* utilizes a series of strategies to antagonize or escape host resistance mechanisms at every stage of infection (Figure 7).

The establishment of a successful infection is dependent on *C. fructicola* invasion, which is the first step of infection and includes the degradation of the plant cell wall and subsequent penetration into the cell. The top 100 up-regulated genes at 24 hpi provide a glimpse into *C. fructicola*–strawberry interactions (Tables S27, S28). Specifically, at this stage, we found that 15 of the top 100 up-regulated *C. fructicola* genes were identified to encode putative effectors, one of which was a chitin–binding protein (CGGC5_3464). The literature shows that the pathogen is likely to initiate its adhesion to the host cells by expressing accessory molecules, such as chitinases and/or chitin-binding proteins (Chaudhuri et al., 2010). Four homologs of HsbA were within the top 100 up-regulated genes at 24 hpi. As previously mentioned HsbA binds to PBSA hydrophobic surfaces and recruits a polyesterase for the degradation of PBSA (Ohtaki et al., 2006). It prompts us to speculate that four homologs of HsbA are involved in attachment to the strawberry surface and the degradation of constituents from the plant epidermis; however, this requires further elucidation. Meanwhile, the host expresses a series of proteins to stabilize the cell wall to cope with *C. fructicola* invasion. Among the top 100 up-regulated strawberry genes, the most prevalent genes (*n* = 5) encode proteins involved in cell wall reinforcement and catabolism. This includes the glycine-rich cell wall structural protein, pectin acetylesterase family protein, wound-induced protein WI12, chitinase and AMP-dependent glycosyl hydrolase. WI12 preferentially accumulates in the cell wall, which suggests that it plays a role in cell wall reinforcement after wounding and during plant development (Yen et al., 2001). Glycine-rich proteins (GRPs) form a major part of the highly extensible and specialized cell walls. However, there has been no genetic evidence indicating a specific function for GRPs (Ringli et al., 2001). Although we did not thoroughly examine the function of these proteins, their high level of expression in our data indicates that the plant cell wall seems to be reinforced to prevent fungal invasion. However, most of the genes encoding cellulose synthase-like proteins, which participate in cellulose biosynthesis and cell wall modification, were down-regulated (Table S15 and Figure 7, upper panel). We conclude that this down-regulation might betargeted by *C. fructicola*, which served to help the fungus break down the cell wall and invade the host cells.

After *C. fructicola* invasion, fungus PAMPs are sensed by host PRRs. A number of strawberry genes encoding RLKs were up-regulated (Figure 7, left panel). In addition, several plant chitinase-encoding genes (*n* = 7) were significantly up-regulated, especially during the late stage of *C. fructicola* infection (Table S29). Chitinases were identified to be induced in various plants after fungal infection (Punja and Zhang, 1993). Furthermore, the antifungal activity of chitinases was shown by large number of transgenic plants raised for over expression of chitinase genes (de las Mercedes Dana et al., 2006). To evade recognition by plant PRRs and subsequent elimination, the fungus utilizes numerous effectors to suppress PTI. Typically, secreted LysM Protein1 (SLP1) can bind to chitin and suppress chitin-induced plant immune responses (Mentlak et al., 2012). Our results show that the SLP1-encoding gene was up-regulated at 72 and 96 hpi. The highly-expressed *SLP1* may play an important role in the suppression of PTI, such as chitin-induced immunity; however, this requires further investigation. A subset of these effectors might be perceived by R proteins resulting in a second layer of host defense called ETI. A total of 10 R genes were significantly up-regulated during *C. fructicola* infection; this suggests that they may play a role in antagonizing effectors, although their functions still need to be characterized (Figure 7 right panel, blue shape).

After recognition of the fungus by PRR and R proteins, the plant initiates downstream anti-fungal innate immunity. In our study, hormone signaling, especially the SA, JA, auxin, ABA, and ET signaling pathways, displayed obvious changes in transcriptional activity (Figure 7, middle panel). The early activation of ABA and auxin signaling, the delayed activation of the SA pathway, and the continuous suppression of the JA and ET pathways might contributed to the deployment of basal defenses and the insufficient induction of the defense system in strawberries. The literature showed that the secreted chorismate mutase protein (Cmu1) of fungus *Ustilago maydis* is proposed to suppress SA-dependent plant defense response (Djamei et al., 2011). Therefore, fungi could formulate efficient strategies against strawberry hormone-related defense systems.

A recent report on the strawberry leaf transcriptomes of “Yanli” and “Benihoppe” cultivars infected with *C. gloeosporioides* consistently showed that PR1 expression was up-regulated after infection, thus supporting the SA pathway response (Wang et al., 2017). Similarly, various defense-related genes, such as those encoding PR proteins, peroxidases, and lipoxygenases, were up-regulated in bean leaf following inoculation with *C*. *lindemuthianum*, which is in accordance with our transcriptomic data (Padder et al., 2016). A dual RNA-seq analysis on *C. graminicola*-maize leaf pathosystem during appressorial maturation, penetration, and colonization revealed that genes encoding secreted proteins, secondary metabolism enzymes, and receptors were over-represented among the differentially expressed genes. Several genes encoding homologs of BAS2, SLP1, and GAS1 were significantly up-regulated following *C. graminicola* infection. The up-regulation of these genes werealsoobserved in the current study, which suggests that these effectors may play important conserved roles in *Colletotrichum* infection (Torres et al., 2016).

In conclusion, the RNA-Seq technique was used to obtain a comprehensive characterization of infection-responsive DEGs from both *C. fructicola* and strawberry. The data revealed the metabolic, host/pathogen-responsive genes, and transcriptional networks associated with *C. fructicola–*strawberry interactions. This study resulted in several novel findings regarding *C. fructicola–*strawberry interactions: (1) an overview of strawberry defense and *C. fructicola* evasion mechanisms during the infection process; (2) a large number of up-regulated fungal genes encode candidate effector proteins during plant invasion; (3) the pivotal role of SA signaling during *C. fructicola* infection; and (4) other plant defense-related genes involved in cell wall fortification and ROS production were differentially regulated in response to *C. fructicola* infection. Future functional studies are needed to confirm the role of these candidate genes in *C. fructicola–*strawberry interactions; in particular, these studies will focus on the *C. fructicola-*specific effector genes and the putative susceptibility-related genes.

## Materials and methods

### Preparation of control and infected samples for host plants and pathogens

The *C. fructicola* isolate (CGMCC3.17371) was obtained from the Shanghai Academy of Agricultural Sciences, China. A susceptible strawberry (*Fragaria* × *ananassa* Duch.) cultivar, “JiuXiang” was used in this study. The plant growth and inoculation protocols have been previously described (Zhang et al., 2016). Briefly, the plants were selected for their uniform size and color and absence of visual defects. Stolon-derived healthy plants with more than 10 fully expanded compound leaves were inoculated in a growth chamber using a spore density of 1 × 10^6^ spores per mL with 0.01% (v/v) Tween 20 in sterile water. Mock inoculations of plants were made using just the Tween 20 water solution. The mature 5th−6th tri-foliate leaves were harvested at 24, 72, and 96 hpi respectively, for RNA-seq analysis, real-time qRT-PCR and qualification of SA, JA, auxin (indole-3-acetic acid [IAA]), and ABA. The strawberry control samples, mock inoculation with water containing Tween-20 were conducted. Five leaves collected from five independent plants at any time point were polled as a biological replicate. Three independent biological replicates were sequenced for each treatment. For the pathogen control samples, the isolate was cultured on potato dextrose agar (PDA) covered with a layer of cellophane. Mycelium (0.2 g) was harvested from each plate, placed in sterile 2 mL centrifuge tubes, and frozen in liquid nitrogen. Three independent mycelial samples were collected for RNA-sequencing.

### Microscopic observation

Leaf samples were collected at 0, 12, 24, 72, and 96 hpi. Aniline blue staining was performed as described previously (Ge and Guest, 2011). Briefly, leaves were decolorized, rinsed, and stained with 0.025% (w/v) aniline blue in lactophenol. The leaves were then rinsed, mounted on glass slides in 50% fresh glycerol, and examined under a Nikon E200 microscope. Images were captured with a Nikon DS-U3 digital camera.

### RNA extraction, construction of illumina library, and sequencing

Total RNA was extracted using RNAiso Plus (Total RNA extraction reagent) (TaKaRa, Otsu, Japan). The samples included mycelium grown in medium, mock inoculation and inoculation leaves at three different time points. RNA purity was verified using a nano spectrophotometer (Implen, Westlake Village, CA, USA). RNA concentrations were measured using a Qubit RNA Assay Kit in a Qubit 2.0 fluorometer (Life Technologies, GrandIsland, NY, USA). RNA integrity was assessed using the RNA Nano 6000 Assay Kit and the Bioanalyzer 2100 system (Agilent Technologies, Wilmington, DE, USA).

Construction of the Illumina library was carried out as previously described (Sun et al., 2016). The cDNA libraries were sequenced on the Illumina HiSeq2500 platform (125 bp paired-end reads) at Shanghai Hanyu Bio-Teche (Shanghai, China). Raw reads were submitted to the NCBI SRA database under accession numbers SRP097590 and SRP099166.

### Data analysis and real-time qRT-PCR

Low quality reads (more than 30% bases with Q < 20) were filtered from the raw reads to obtain high-quality reads. Genomes of the strawberry (*F*. *vesca*, http://www.rosaceae.org/species/fragaria/fragaria_vesca/genome_v1.0) and *C. fructicola* Nara gc5 isolate (ftp://ftp.ncbi.nlm.nih.gov/genomes/genbank/fungi/Colletotrichum_gloeosporioides/) were used as reference sequences. After trimming low-quality bases (Q < 20) from the 5′ and 3′ ends of the remaining reads, the resulting high-quality reads were mapped tothe proper reference sequence using Top Hat v1.3.0 (Trapnell et al., 2009), which allows for one mismatch. Gene expression levels in the RNA-seq analysis were measured as reads per kilobase of exon model per million mapped reads (RPKM). Differentially expressed genes were called via DEGeq package and Cuffdiff which was developed with special attention to cope with biological variance (Anders and Huber, 2010). The MARS model (MA-plot-based method with the random sampling model) in the DEGseq package was used to calculate the expression abundance of each gene via pairwise comparisons (Mycelium vs. fungus *in planta* and mock inoculation vs. infected). The FDR (false discovery rate) method was used to determine the threshold *p*-value for multiple testing. Genes whose expression differences were significant at FDR < 0.001, and |Normalized Fold change|>2 were identified as differentially expressed (Anders and Huber, 2010). The analysis mapped all DEGs to GO terms in the database (http://www.geneontology.org/) to investigate gene ontology enrichment. The PFAM server (http://pfam.sanger.ac.uk/) was used to verify the function of DEGs in *C. fructicola*. PCWDEs were classified using the dbCAN HMMer-based classification system (Yin et al., 2012), applying an *E*-value cut-off of 10E-5. Potential secondary metabolite clusters were identified using SMURF (Khaldi et al., 2010).

Quantitativereal-time PCR (qRT-PCR) was carried out as previously described (Rudd et al., 2015). All primers are listed in the supporting information (Table S1). In brief, total RNA (1 μg) from mycelium, mock inoculated strawberry leaves and *C. fructicola*-infected strawberry leaves (24, 72, and 96 hpi) was reverse transcribed to cDNA with an oligo(dT) primer using SuperScript III (Invitrogen, Carlsbad, CA 92008, USA) according to the manufacturer's instructions. A standard PCR assay for each primer was conducted. The cDNA samples for which one specific band at the desired size was observed were used. The Premix Ex Taq (Perfect Real Time) kit (Takara, Dalian, China, DRR041A) was used for qRT-PCR on an ABI 7500 Real-Time Cycler (Applied Biosystems, Waltham, MA, USA). qRT-PCR was performed with 20 ng of cDNA, 10 μL of enzyme mixture from the kit, 10 pmol of sense primers and 10 pmol of antisense primers in a final volume of 20 μL. The PCR cycles are as follows: 95°C for 15 s, followed by 40 cycles of 95°C for 5 s, 55°C for 10 s and 72°C for 15 s. At the end of the reaction, melt curves were run for all primer pairs in order to check for dimerization. PCR efficiency was >1.85 and amplifications generated single expected amplicons with single, sharp fusion curves. The expression levels of target mRNA were normalized to that of reference genes and calculated by the comparative CT method as described (Schmittgen and Livak, 2008). Each plate was repeated thrice in independent runs for all reference and selected genes. Data were analyzed using the Applied Biosystems 7500 software version 2.0.1.

### *C. fructicola* secretome prediction and analysis pipeline

We developed a pipeline to predict and classify effectors of *C. fructicola* as previously described (Haddadi et al., 2016). Pfam (Finn et al., 2014) was used to predict biological functions enriched in the secretome of *C. fructicola*. The candidate effectors were then further evaluated with the EffectorP prediction tool (effectorp.csiro.au) (Sperschneider et al., 2016). N- and O-linked glycosylation sites were predicted using the NetNGlyc 1.0 (http://www.cbs.dtu.dk/services/NetNGlyc) and NetOGlyc 2.0 (http://www.cbs.dtu.dk/services/NetOGlyc) servers, respectively.

### *Agrobacterium tumefaciens* infiltration assays

Genes for effector candidates were amplified from the *C. fructicola*-strawberry cDNA library using the corresponding primers (Table S1). The PCR products were subcloned between the cauliflower mosaic virus (CaMV) 35S promoter and the green fluorescent protein (S65T mutant, sGFP) reporter gene in pBluscript. The constructs were confirmed by sequencing at Invitrogen. *Agrobacterium tumefaciens* infiltration assays were performed according to previously described methods (Wang et al., 2011)with minor modifications. Briefly, *Agrobacterium* were grown in Luria-Bertani media plus 50 μg/mL kanamycin for 48 h, harvested, and washed with 10 mM MgCl_2_ three times, re-suspended in 10 mM MgCl_2_ to a final OD_600_ of 0.8–1.5, and then incubated at room temperature for 3 h prior to infiltration. Leaves of 4–6 weeks old *N*. *benthamiana* were infiltrated with a needleless syringe and photographed 3–5 days after infiltration. For the suppression of Bax-mediated cell death, the *A. tumefaciens-containing* Bax gene and *A. tumefaciens* strain carrying the individual effector genes were infiltrated in the same spot. *A. tumefaciens* strains carrying Bax, GFP or both Bax and GFP genes, were infiltrated in parallel as controls. The experiment was repeated three times with each assay consisting of three plants each with three leaves inoculation.

### Determination of four endogenous phyto-hormones

The strawberry leaf samples were sent to the Analysis and Testing Center at Beijing Forestry University (Beijing) for ultra-performance liquid chromatography-tandem mass spectrometry (UPLC-MS/MS) analysis. Free SA, JA, auxin (indole-3-acetic acid [IAA]) and ABA were quantified referring to Pan et al. (2010). In brief, free hormones were extracted from ~100 mg of leaf tissues first by 90% methanol. The crude plant extracts were then re-dissolved by methanol-water (1:1 in volume) and filtered through 0.24 μm infiltration head before analysis. The UPLC/MS system consisted of an Agilent 1260 detector and ABQ trap 5500 system. The Agilent C18 main column (4.6 × 50 mm, 1.8 μm) was used for separation of different hormonal components. The internal standards were purchased from Sigma Aldrich Company.

### Statistical analysis

The differentially expressed genes (DEGs) were subjected to GO enrichment analysis. DEGs were first mapped to GO terms using a standard database (http://www.geneontology.org/); gene numbers for each term were calculated, and GO terms significantly enriched in DEGs compared to the background genome were determine with a hypergeometric test. All calculated *p*-values were then subjected to Bonferroni correction, using a corrected *p* ≤ 0.05 as the threshold. GO terms that fulfilled this criterion were defined as significantly enriched in DEGs. For phytohormones determination, the data were analyzed by ANOVA, with *p* < 0.05 considered statistically significant.

## Author contributions

The study was conceived by KD, QG, and LZ. LZ prepared the *C. fructicola* isolate and plant materials and performed the experiments. LZ, XH, and XZ performed the bioinformatics analysis. CH performed the experiments of *Agrobacterium tumefaciens* infiltration. Q-YZ measured plant hormones in all samples. LZ, KD, and QG prepared the manuscript. All authors contributed to revising the manuscript. All authors had read and approved the final manuscript.

### Conflict of interest statement

The authors declare that the research was conducted in the absence of any commercial or financial relationships that could be construed as a potential conflict of interest.

## References

[B1] AbuQamar S. Chen X. Dhawan R. Bluhm B. Salmeron J. Lam S.. (2006). Expression profiling and mutant analysis reveals complex regulatory networks involved in Arabidopsis response to Botrytis infection. Plant J. 48, 28–44. 10.1111/j.1365-313X.2006.02849.x 16925600

[B2] Alayon-Luaces P. Pagano E. A. Mroginski L. A. Sozzi G. O. (2008). Four glycoside hydrolases are differentially modulated by auxins, cytokinins, abscisic acid and gibberellic acid in apple fruit callus cultures. Plant Cell Tiss. Organ. Cult. 95, 257–263. 10.1007/s11240-008-9438-1

[B3] Alkan N. Friedlander G. Ment D. Prusky D. Fluhr R. (2015). Simultaneous transcriptome analysis of *Colletotrichum gloeosporioides* and tomato fruit pathosystem reveals novel fungal pathogenicity and fruit defense strategies. New Phytol. 205, 801–815. 10.1111/nph.13087 25377514

[B4] Almeida N. F. Krezdorn N. Rotter B. Winter P. Rubiales D. Vaz Patto M. C. (2015). *Lathyrus sativus* transcriptome resistance response to *Ascochyta lathyri* investigated by deepSuperSAGE analysis. Front. Plant Sci. 6:178. 10.3389/fpls.2015.00178 25852725 PMC4367168

[B5] Amil-Ruiz F. Blanco-Portales R. Munoz-Blanco J. Caballero J. L. (2011). The strawberry plant defense mechanism: a molecular review. Plant Cell Physiol. 52, 1873–1903. 10.1093/pcp/pcr136 21984602

[B6] Anders S. Huber W. (2010). Differential expression analysis for sequence count data. Genome Biol. 11:R106. 10.1186/gb-2010-11-10-r106 20979621 PMC3218662

[B7] Anwar A. K. Ding S. S. (2004). Molecular cloning, characterization, and expression analysis of two class II chitinase genes from the strawberry plant. Plant Sci. 166, 753–762. 10.1016/j.plantsci.2003.11.015

[B8] Apel K. Hirt H. (2004). Reactive oxygen species: metabolism, oxidative stress, and signal transduction. Annu. Rev. Plant Biol. 55, 373–399. 10.1146/annurev.arplant.55.031903.141701 15377225

[B9] Audenaert K. De Meyer G. B. Hofte M. M. (2002). Abscisic acid determines basal susceptibility of tomato to *Botrytis cinerea* and suppresses salicylic acid-dependent signaling mechanisms. Plant Physiol. 128, 491–501. 10.1104/pp.010605 11842153 PMC148912

[B10] Bari R. Jones J. D. (2009). Role of plant hormones in plant defence responses. Plant Mol. Biol. 69, 473–488. 10.1007/s11103-008-9435-0 19083153

[B11] Bell E. Creelman R. A. Mullet J. E. (1995). A chloroplast lipoxygenase is required for wound-induced jasmonic acid accumulation in Arabidopsis. Proc. Natl. Acad. Sci. U.S.A. 92, 8675–8679. 10.1073/pnas.92.19.8675 7567995 PMC41029

[B12] Bellincampi D. Cervone F. Lionetti V. (2014). Plant cell wall dynamics and wall-related susceptibility in plant-pathogen interactions. Front. Plant Sci. 5:228. 10.3389/fpls.2014.00228 24904623 PMC4036129

[B13] Bertini L. Leonardi L. Caporale C. (2003). Pathogen-responsive wheat PR4 genes are induced by activators of systemic acquired resistance and wounding. Plant Sci. 164, 1067–1078. 10.1016/S0168-9452(03)00112-2

[B14] Birkenbihl R. P. Diezel C. Somssich I. E. (2012). Arabidopsis WRKY33 is a key transcriptional regulator of hormonal and metabolic responses toward *Botrytis cinerea* infection. Plant Physiol. 159, 266–285. 10.1104/pp.111.192641 22392279 PMC3375964

[B15] Bolton M. D. van Esse H. P. Vossen J. H. de Jonge R. Stergiopoulos I. Stulemeijer I. J.. (2008). The novel *Cladosporium fulvum* lysin motif effector Ecp6 is a virulence factor with orthologues in other fungal species. Mol. Microbiol. 69, 119–136. 10.1111/j.1365-2958.2008.06270.x 18452583

[B16] Caño-Delgado A. Penfield S. Smith C. Catley M. Bevan M. (2003). Reduced cellulose synthesis invokes lignification and defense responses in *Arabidopsis thaliana*. Plant J. 34, 351–362. 10.1046/j.1365-313X.2003.01729.x 12713541

[B17] Chaudhuri S. Bruno J. C. Alonzo F. 3rd Xayarath B. Cianciotto N. P. Freitag N. E. (2010). Contribution of chitinases to *Listeria monocytogenes* pathogenesis. Appl. Environ. Microbiol. 76, 7302–7305. 10.1128/AEM.01338-10 20817810 PMC2976247

[B18] Chisholm S. T. Coaker G. Day B. Staskawicz B. J. (2006). Host-microbe interactions: shaping the evolution of the plant immune response. Cell 124, 803–814. 10.1016/j.cell.2006.02.008 16497589

[B19] Choquer M. Lee M. H. Bau H. J. Chung K. R. (2007). Deletion of a MFS transporter-like gene in *Cercospora nicotianae* reduces cercosporin toxin accumulation and fungal virulence. FEBS Lett. 581, 489–494. 10.1016/j.febslet.2007.01.011 17250832

[B20] Chumley F. G. (1990). Genetic analysis of melanin-deficient, nonpathogenic mutants of *Magnaporthe grisea*. Mol. Plant Microbe Interact. 3:135.

[B21] Clarke J. D. Volko S. M. Ledford H. Ausubel F. M. Dong X. (2000). Roles of salicylic acid, jasmonic acid, and ethylene in cpr-induced resistance in arabidopsis. Plant Cell 12, 2175–2190. 10.1105/tpc.12.11.2175 11090217 PMC150166

[B22] Coleman J. J. Mylonakis E. (2009). Efflux in fungi: la piece de resistance. PLoS Pathog. 5:e1000486. 10.1371/journal.ppat.1000486 19557154 PMC2695561

[B23] Coleman J. J. White G. J. Rodriguez-Carres M. Vanetten H. D. (2011). An ABC transporter and a cytochrome P450 of *Nectria haematococca* MPVI are virulence factors on pea and are the major tolerance mechanisms to the phytoalexin pisatin. Mol. Plant Microbe Interact. 24, 368–376. 10.1094/MPMI-09-10-0198 21077772

[B24] Crešnar B. Petric S. (2011). Cytochrome P450 enzymes in the fungal kingdom. Biochim. Biophys. Acta 1814, 29–35. 10.1016/j.bbapap.2010.06.020 20619366

[B25] Dai X. You C. Chen G. Li X. Zhang Q. Wu C. (2011). OsBC1L4 encodes a COBRA-like protein that affects cellulose synthesis in rice. Plant Mol. Biol. 75, 333–345. 10.1007/s11103-011-9730-z 21264494

[B26] Davin L. B. Wang H. B. Crowell A. L. Bedgar D. L. Martin D. M. Sarkanen S.. (1997). Stereoselective bimolecular phenoxy radical coupling by an auxiliary (dirigent) protein without an active center. Science 275, 362–366. 10.1126/science.275.5298.362 8994027

[B27] de Jonge R. Thomma B. P. (2009). Fungal LysM effectors: extinguishers of host immunity? Trends Microbiol. 17, 151–157. 10.1016/j.tim.2009.01.002 19299132

[B28] de Jonge R. van Esse H. P. Kombrink A. Shinya T. Desaki Y. Bours R.. (2010). Conserved fungal LysM effector Ecp6 prevents chitin-triggered immunity in plants. Science 329, 953–955. 10.1126/science.1190859 20724636

[B29] de las Mercedes Dana M. Pintor-Toro J. A. Cubero B. (2006). Transgenic tobacco plants overexpressing chitinases of fungal origin show enhanced resistance to biotic and abiotic stress agents. Plant Physiol. 142, 722–730. 10.1104/pp.106.086140 16891545 PMC1586035

[B30] Del Sorbo G. Schoonbeek H. De Waard M. A. (2000). Fungal transporters involved in efflux of natural toxic compounds and fungicides. Fungal Genet. Biol. 30, 1–15. 10.1006/fgbi.2000.1206 10955904

[B31] Denance N. Sanchez-Vallet A. Goffner D. Molina A. (2013). Disease resistance or growth: the role of plant hormones in balancing immune responses and fitness costs. Front. Plant Sci. 4:155. 10.3389/fpls.2013.00155 23745126 PMC3662895

[B32] Djamei A. Schipper K. Rabe F. Ghosh A. Vincon V. Kahnt J.. (2011). Metabolic priming by a secreted fungal effector. Nature 478, 395–398. 10.1038/nature10454 21976020

[B33] Doares S. H. Narvaez-Vasquez J. Conconi A. Ryan C. A. (1995). Salicylic acid inhibits synthesis of proteinase inhibitors in tomato leaves induced by systemin and jasmonic acid. Plant Physiol. 108, 1741–1746. 10.1104/pp.108.4.1741 12228577 PMC157556

[B34] Doehlemann G. Requena N. Schaefer P. Brunner F. O'Connell R. Parker J. E. (2014). Reprogramming of plant cells by filamentous plant-colonizing microbes. New Phytol. 204, 803–814. 10.1111/nph.12938 25539003

[B35] Eisenman H. C. Casadevall A. (2012). Synthesis and assembly of fungal melanin. Appl. Microbiol. Biotechnol. 93, 931–940. 10.1007/s00253-011-3777-2 22173481 PMC4318813

[B36] Esquerré-Tugayé M. T. Boudart G. Dumas B. (2000). Cell wall degrading enzymes, inhibitory proteins, and oligosaccharides participate in the molecular dialogue between plants and pathogens. Plant Physiol. Biochem. 38, 157–163. 10.1016/S0981-9428(00)00161-3

[B37] Eulgem T. Somssich I. E. (2007). Networks of WRKY transcription factors in defense signaling. Curr. Opin. Plant Biol. 10, 366–371. 10.1016/j.pbi.2007.04.020 17644023

[B38] Fankhauser N. Mäser P. (2005). Identification of GPI anchor attachment signals by a Kohonen self-organizing map. Bioinformatics 21, 1846–1852. 10.1093/bioinformatics/bti299 15691858

[B39] Finn R. D. Bateman A. Clements J. Coggill P. Eberhardt R. Y. Eddy S. R.. (2014). Pfam: the protein families database. Nucleic Acids Res. 42, D222–D230. 10.1093/nar/gkt1223 24288371 PMC3965110

[B40] Fitzpatrick A. H. Shrestha N. Bhandari J. Crowell D. N. (2011). Roles for farnesol and ABA in Arabidopsis flower development. Plant Signal. Behav. 6, 1189–1191. 10.4161/psb.6.8.15772 21758018 PMC3260718

[B41] Gan P. Ikeda K. Irieda H. Narusaka M. O'Connell R. J. Narusaka Y.. (2013). Comparative genomic and transcriptomic analyses reveal the hemibiotrophic stage shift of Colletotrichum fungi. New Phytol. 197, 1236–1249. 10.1111/nph.12085 23252678

[B42] Gan P. Nakata N. Suzuki T. Shirasu K. (2017). Markers to differentiate species of anthracnose fungi identify *Colletotrichum fructicola* as the predominant virulent species in strawberry plants in Chiba Prefecture of Japan. J. Gen. Plant Pathol. 83, 14–22. 10.1007/s10327-016-0689-0

[B43] Ge Y. Guest D. I. (2011). Light and scanning electron microscopy studies on the infection process of melon leaves by *Colletotrichum lagenarium*. Physiol. Mol. Plant Pathol. 76, 67–74. 10.1016/j.pmpp.2011.06.001

[B44] Glazebrook J. (2005). Contrasting mechanisms of defense against biotrophic and necrotrophic pathogens. Annu. Rev. Phytopathol. 43, 205–227. 10.1146/annurev.phyto.43.040204.135923 16078883

[B45] Göhre V. Robatzek S. (2008). Breaking the barriers: microbial effector molecules subvert plant immunity. Annu. Rev. Phytopathol. 46, 189–215. 10.1146/annurev.phyto.46.120407.110050 18422429

[B46] Gupta V. Willits M. G. Glazebrook J. (2000). *Arabidopsis thaliana* EDS4 contributes to salicylic acid (SA)-dependent expression of defense responses: evidence for inhibition of jasmonic acid signaling by SA. Mol. Plant Microbe Interact. 13, 503–511. 10.1094/MPMI.2000.13.5.503 10796016

[B47] Guyon K. Balague C. Roby D. Raffaele S. (2014). Secretome analysis reveals effector candidates associated with broad host range necrotrophy in the fungal plant pathogen *Sclerotinia sclerotiorum*. BMC Genomics 15:336. 10.1186/1471-2164-15-336 24886033 PMC4039746

[B48] Haddadi P. Ma L. Wang H. Borhan M. H. (2016). Genome-wide transcriptomic analyses provide insights into the lifestyle transition and effector repertoire of *Leptosphaeria maculans* during the colonization of *Brassica napus* seedlings. Mol. Plant Pathol. 17, 1196–1210. 10.1111/mpp.12356 26679637 PMC6638455

[B49] Hamann T. (2012). Plant cell wall integrity maintenance as an essential component of biotic stress response mechanisms. Front. Plant Sci. 3:77. 10.3389/fpls.2012.00077 22629279 PMC3355559

[B50] Han Y. C. Zeng X. G. Xiang F. Y. Ren L. Chen F. Y. Gu Y. C. (2016). Distribution and characteristics of colletotrichum spp. associated with anthracnose of strawberry in Hubei, China. Plant Dis. 100, 996–1006. 10.1094/PDIS-09-15-1016-RE 30686149

[B51] Henz G. P. Boiteux L. S. Lopes C. A. (1992). Outbreak of strawberry anthracnose caused by *Colletotrichum acutatum* in Central Brazil. Plant Dis. 76:212.

[B52] Hong K. Gong D. Zhang L. Hu H. Jia Z. Gu H.. (2016). Transcriptome characterization and expression profiles of the related defense genes in postharvest mango fruit against *Colletotrichum gloeosporioides*. Gene 576(1 Pt 2), 275–283. 10.1016/j.gene.2015.10.041 26496007

[B53] Jensen M. K. Hagedorn P. H. de Torres-Zabala M. Grant M. R. Rung J. H. Collinge D. B.. (2008). Transcriptional regulation by an NAC (NAM-ATAF1,2-CUC2) transcription factor attenuates ABA signalling for efficient basal defence towards *Blumeria graminis* f. sp. hordei in Arabidopsis. Plant J. 56, 867–880. 10.1111/j.1365-313X.2008.03646.x 18694460

[B54] Kaku H. Nishizawa Y. Ishii-Minami N. Akimoto-Tomiyama C. Dohmae N. Takio K.. (2006). Plant cells recognize chitin fragments for defense signaling through a plasma membrane receptor. Proc. Natl. Acad. Sci. U.S.A. 103, 11086–11091. 10.1073/pnas.0508882103 16829581 PMC1636686

[B55] Katagiri F. Tsuda K. (2010). Understanding the plant immune system. Mol. Plant Microbe Interact. 23, 1531–1536. 10.1094/MPMI-04-10-0099 20653410

[B56] Ke X. Yin Z. Song N. Dai Q. Voegele R. T. Liu Y.. (2014). Transcriptome profiling to identify genes involved in pathogenicity of *Valsa mali* on apple tree. Fungal Genet. Biol. 68, 31–38. 10.1016/j.fgb.2014.04.004 24747070

[B57] Khaldi N. Seifuddin F. T. Turner G. Haft D. Nierman W. C. Wolfe K. H.. (2010). SMURF: genomic mapping of fungal secondary metabolite clusters. Fungal Genet. Biol. 47, 736–741. 10.1016/j.fgb.2010.06.003 20554054 PMC2916752

[B58] Kim S. Ahn I. P. Rho H. S. Lee Y. H. (2005). MHP1, a *Magnaporthe grisea* hydrophobin gene, is required for fungal development and plant colonization. Mol. Microbiol. 57, 1224–1237. 10.1111/j.1365-2958.2005.04750.x 16101997

[B59] Kleemann J. Rincon-Rivera L. J. Takahara H. Neumann U. Ver Loren van Themaat E. van der Does H. C.. (2012). Sequential delivery of host-induced virulence effectors by appressoria and intracellular hyphae of the phytopathogen *Colletotrichum higginsianum*. PLoS Pathog. 8:e1002643. 10.1371/journal.ppat.1002643 22496661 PMC3320591

[B60] Koeck M. Hardham A. R. Dodds P. N. (2011). The role of effectors of biotrophic and hemibiotrophic fungi in infection. Cell. Microbiol. 13, 1849–1857. 10.1111/j.1462-5822.2011.01665.x 21848815 PMC3218205

[B61] Kubo Y. Takano Y. (2013). Dynamics of infection-related morphogenesis and pathogenesis in *Colletotrichum orbiculare*. J. Gen. Plant Pathol. 79, 233–242. 10.1007/s10327-013-0451-9

[B62] Lee D. Ellard M. Wanner L. A. Davis K. R. Douglas C. J. (1995). The *Arabidopsis thaliana* 4-coumarate:CoA ligase (4CL) gene: stress and developmentally regulated expression and nucleotide sequence of its cDNA. Plant Mol. Biol. 28, 871–884. 10.1007/BF00042072 7640359

[B63] Lee S. H. Han Y. K. Yun S. H. Lee Y. W. (2009). Roles of the glyoxylate and methylcitrate cycles in sexual development and virulence in the cereal pathogen Gibberella zeae. Eukaryotic Cell 8, 1155–1164. 10.1128/EC.00335-08 19525419 PMC2725564

[B64] Li J. Zhang Q. Y. Gao Z. H. Wang F. Duan K. Ye Z. W.. (2013). Genome-wide identification and comparative expression analysis of NBS-LRR-encoding genes upon *Colletotrichum gloeosporioides* infection in two ecotypes of *Fragaria vesca*. Gene 527, 215–227. 10.1016/j.gene.2013.06.008 23806759

[B65] Liu L. Sonbol F. M. Huot B. Gu Y. Withers J. Mwimba M.. (2016). Salicylic acid receptors activate jasmonic acid signalling through a non-canonical pathway to promote effector-triggered immunity. Nat. Commun. 7:13099. 10.1038/ncomms13099 27725643 PMC5062614

[B66] Llorente F. Muskett P. Sanchez-Vallet A. Lopez G. Ramos B. Sanchez-Rodriguez C.. (2008). Repression of the auxin response pathway increases Arabidopsis susceptibility to necrotrophic fungi. Mol. Plant 1, 496–509. 10.1093/mp/ssn025 19825556

[B67] Lo Presti L. Lanver D. Schweizer G. Tanaka S. Liang L. Tollot M.. (2015). Fungal effectors and plant susceptibility. Annu. Rev. Plant Biol. 66, 513–545. 10.1146/annurev-arplant-043014-114623 25923844

[B68] Lorenzo O. Piqueras R. Sanchez-Serrano J. J. Solano R. (2003). Ethylene response factor1 integrates signals from ethylene and jasmonate pathways in plant defense. Plant Cell 15, 165–178. 10.1105/tpc.007468 12509529 PMC143489

[B69] MacKenzie S. J. Legard D. E. Timmer L. W. Chandler C. K. Peres N. A. (2006). Resistance of strawberry cultivars to crown rot caused by *Colletotrichum gloeosporioides* isolates from Florida is nonspecific. Plant Dis. 90, 1091–1097. 10.1094/PD-90-1091 30781305

[B70] Malinovsky F. G. Fangel J. U. Willats W. G. (2014). The role of the cell wall in plant immunity. Front. Plant Sci. 5:178. 10.3389/fpls.2014.00178 24834069 PMC4018530

[B71] Mentlak T. A. Kombrink A. Shinya T. Ryder L. S. Otomo I. Saitoh H.. (2012). Effector-mediated suppression of chitin-triggered immunity by *Magnaporthe oryzae* is necessary for rice blast disease. Plant Cell 24, 322–335. 10.1105/tpc.111.092957 22267486 PMC3289562

[B72] Miya A. Albert P. Shinya T. Desaki Y. Ichimura K. Shirasu K.. (2007). CERK1, a LysM receptor kinase, is essential for chitin elicitor signaling in Arabidopsis. Proc. Natl. Acad. Sci. U.S.A. 104, 19613–19618. 10.1073/pnas.0705147104 18042724 PMC2148337

[B73] Moore J. W. Loake G. J. Spoel S. H. (2011). Transcription dynamics in plant immunity. Plant Cell 23, 2809–2820. 10.1105/tpc.111.087346 21841124 PMC3180793

[B74] Mou Z. Fan W. Dong X. (2003). Inducers of plant systemic acquired resistance regulate NPR1 function through redox changes. Cell 113, 935–944. 10.1016/S0092-8674(03)00429-X 12837250

[B75] Nam M. H. Park M. S. Lee H. D. Yu S. H. (2013). Taxonomic re-evaluation of *Colletotrichum gloeosporioides* isolated from strawberry in Korea. Plant Pathol. J. 29, 317–322. 10.5423/PPJ.NT.12.2012.0188 25288958 PMC4174798

[B76] Nelson D. R. (1999). Cytochrome P450 and the individuality of species. Arch. Biochem. Biophys. 369, 1–10. 10.1006/abbi.1999.1352 10462435

[B77] Nicaise V. Roux M. Zipfel C. (2009). Recent advances in PAMP-triggered immunity against bacteria: pattern recognition receptors watch over and raise the alarm. Plant Physiol. 150, 1638–1647. 10.1104/pp.109.139709 19561123 PMC2719144

[B78] Niderman T. Genetet I. Bruyere T. Gees R. Stintzi A. Legrand M.. (1995). Pathogenesis-related PR-1 proteins are antifungal. Isolation and characterization of three 14-kilodalton proteins of tomato and of a basic PR-1 of tobacco with inhibitory activity against *Phytophthora infestans*. Plant Physiol. 108, 17–27. 10.1104/pp.108.1.17 7784503 PMC157301

[B79] O'Connell R. Herbert C. Sreenivasaprasad S. Khatib M. Esquerre-Tugaye M. T. Dumas B. (2004). A novel Arabidopsis-Colletotrichum pathosystem for the molecular dissection of plant-fungal interactions. Mol. Plant Microbe Interact. 17, 272–282. 10.1094/MPMI.2004.17.3.272 15000394

[B80] O'Connell R. J. Thon M. R. Hacquard S. Amyotte S. G. Kleemann J. Torres M. F.. (2012). Lifestyle transitions in plant pathogenic Colletotrichum fungi deciphered by genome and transcriptome analyses. Nat. Genet. 44, 1060–1065. 10.1038/ng.2372 22885923 PMC9754331

[B81] Ohtaki S. Maeda H. Takahashi T. Yamagata Y. Hasegawa F. Gomi K.. (2006). Novel hydrophobic surface binding protein, HsbA, produced by *Aspergillus oryzae*. Appl. Environ. Microbiol. 72, 2407–2413. 10.1128/AEM.72.4.2407-2413.2006 16597938 PMC1449009

[B82] Oliveira-Garcia E. Valent B. (2015). How eukaryotic filamentous pathogens evade plant recognition. Curr. Opin. Microbiol. 26, 92–101. 10.1016/j.mib.2015.06.012 26162502

[B83] Padder B. A. Kamfwa K. Awale H. E. Kelly J. D. (2016). Transcriptome profiling of the *Phaseolus vulgaris*- *Colletotrichum lindemuthianum* pathosystem. PLoS ONE 11:e0165823. 10.1371/journal.pone.0165823 27829044 PMC5102369

[B84] Pan X. Welti R. Wang X. (2010). Quantitative analysis of major plant hormones in crude plant extracts by high-performance liquid chromatography-mass spectrometry. Nat. Protoc. 5, 986–992. 10.1038/nprot.2010.37 20448544

[B85] Perfect S. E. Hughes H. B. O'Connell R. J. Green J. R. (1999). Colletotrichum: a model genus for studies on pathology and fungal-plant interactions. Fungal Genet. Biol. 27, 186–198. 10.1006/fgbi.1999.1143 10441444

[B86] Petersen T. N. Brunak S. von Heijne G. Nielsen H. (2011). SignalP 4.0: discriminating signal peptides from transmembrane regions. Nat. Methods 8, 785–786. 10.1038/nmeth.1701 21959131

[B87] Pieterse C. M. Van der Does D. Zamioudis C. Leon-Reyes A. Van Wees S. C. (2012). Hormonal modulation of plant immunity. Annu. Rev. Cell Dev. Biol. 28, 489–521. 10.1146/annurev-cellbio-092910-154055 22559264

[B88] Punja Z. K. Zhang Y. Y. (1993). Plant chitinases and their roles in resistance to fungal diseases. J. Nematol. 25, 526–540. 19279806 PMC2619419

[B89] Ringli C. Keller B. Ryser U. (2001). Glycine-rich proteins as structural components of plant cell walls. Cell. Mol. Life Sci. 58, 1430–1441. 10.1007/PL00000786 11693524 PMC11337278

[B90] Rudd J. J. Kanyuka K. Hassani-Pak K. Derbyshire M. Andongabo A. Devonshire J.. (2015). Transcriptome and metabolite profiling of the infection cycle of *Zymoseptoria tritici* on wheat reveals a biphasic interaction with plant immunity involving differential pathogen chromosomal contributions and a variation on the hemibiotrophic lifestyle definition. Plant Physiol. 167, 1158–1185. 10.1104/pp.114.255927 25596183 PMC4348787

[B91] Sato M. Tsuda K. Wang L. Coller J. Watanabe Y. Glazebrook J.. (2010). Network modeling reveals prevalent negative regulatory relationships between signaling sectors in Arabidopsis immune signaling. PLoS Pathog. 6:e1001011. 10.1371/journal.ppat.1001011 20661428 PMC2908620

[B92] Schaller A. Stintzi A. (2009). Enzymes in jasmonate biosynthesis - structure, function, regulation. Phytochemistry 70, 1532–1538. 10.1016/j.phytochem.2009.07.032 19703696

[B93] Schmittgen T. D. Livak K. J. (2008). Analyzing real-time PCR data by the comparative C(T) method. Nat. Protoc. 3, 1101–1108. 10.1038/nprot.2008.73 18546601

[B94] Shakeel S. N. Wang X. Binder B. M. Schaller G. E. (2013). Mechanisms of signal transduction by ethylene: overlapping and non-overlapping signalling roles in a receptor family. AoB Plants 5:plt010. 10.1093/aobpla/plt010 23543258 PMC3611092

[B95] Shin J. Y. Bui D. C. Lee Y. Nam H. Jung S. Fang M.. (2017). Functional characterization of cytochrome P450 monooxygenases in the cereal head blight fungus *Fusarium graminearum*. Environ. Microbiol. 19, 2053–2067. 10.1111/1462-2920.13730 28296081

[B96] Shulaev V. Sargent D. J. Crowhurst R. N. Mockler T. C. Folkerts O. Delcher A. L.. (2011). The genome of woodland strawberry (*Fragaria vesca*). Nat. Genet. 43, 109–116. 10.1038/ng.740 21186353 PMC3326587

[B97] Sperschneider J. Gardiner D. M. Dodds P. N. Tini F. Covarelli L. Singh K. B.. (2016). EffectorP: predicting fungal effector proteins from secretomes using machine learning. New Phytol. 210, 743–761. 10.1111/nph.13794 26680733

[B98] Stefanato F. L. Abou-Mansour E. Buchala A. Kretschmer M. Mosbach A. Hahn M.. (2009). The ABC transporter BcatrB from *Botrytis cinerea* exports camalexin and is a virulence factor on *Arabidopsis thaliana*. Plant J. 58, 499–510. 10.1111/j.1365-313X.2009.03794.x 19154205

[B99] Strijbis K. Distel B. (2010). Intracellular acetyl unit transport in fungal carbon metabolism. Eukaryot. Cell 9, 1809–1815. 10.1128/EC.00172-10 20889721 PMC3008284

[B100] Sun Y. Guo C. Y. Wang D. D. Li X. F. Xiao L. Zhang X. (2016). Transcriptome analysis reveals the molecular mechanisms underlying growth superiority in a novel grouper hybrid (*Epinephelus fuscogutatus* female symbol x *E. lanceolatusmale* symbol). BMC Genet. 17:24 10.1186/s12863-016-0328-y 26785614 PMC4719697

[B101] Talbot N. J. Ebbole D. J. Hamer J. E. (1993). Identification and characterization of MPG1, a gene involved in pathogenicity from the rice blast fungus *Magnaporthe grisea*. Plant Cell 5, 1575–1590. 10.1105/tpc.5.11.1575 8312740 PMC160387

[B102] Talbot N. J. Kershaw M. J. Wakley G. E. De Vries O. Wessels J. Hamer J. E. (1996). MPG1 encodes a fungal hydrophobin involved in surface interactions during infection-related development of *Magnaporthe grisea*. Plant Cell 8, 985–999. 10.1105/tpc.8.6.985 12239409 PMC161153

[B103] Torres M. F. Ghaffari N. Buiate E. A. Moore N. Schwartz S. Johnson C. D.. (2016). A *Colletotrichum graminicola* mutant deficient in the establishment of biotrophy reveals early transcriptional events in the maize anthracnose disease interaction. BMC Genomics 17:202. 10.1186/s12864-016-2546-0 26956617 PMC4782317

[B104] Trapnell C. Pachter L. Salzberg S. L. (2009). TopHat: discovering splice junctions with RNA-Seq. Bioinformatics 25, 1105–1111. 10.1093/bioinformatics/btp120 19289445 PMC2672628

[B105] Tucker S. L. Talbot N. J. (2001). Surface attachment and pre-penetration stage development by plant pathogenic fungi. Annu. Rev. Phytopathol. 39, 385–417. 10.1146/annurev.phyto.39.1.385 11701871

[B106] van Loon L. C. Rep M. Pieterse C. M. (2006). Significance of inducible defense-related proteins in infected plants. Annu. Rev. Phytopathol. 44, 135–162. 10.1146/annurev.phyto.44.070505.143425 16602946

[B107] Verma V. Ravindran P. Kumar P. P. (2016). Plant hormone-mediated regulation of stress responses. BMC Plant Biol. 16:86. 10.1186/s12870-016-0771-y 27079791 PMC4831116

[B108] Wang F. Zhang F. Chen M. Liu Z. Zhang Z. Fu J.. (2017). Comparative transcriptomics reveals differential gene expression related to *Colletotrichum gloeosporioides* resistance in the octoploid strawberry. Front. Plant Sci. 8:779. 10.3389/fpls.2017.00779 28555149 PMC5430163

[B109] Wang Q. Han C. Ferreira A. O. Yu X. Ye W. Tripathy S.. (2011). Transcriptional programming and functional interactions within the *Phytophthora sojae* RXLR effector repertoire. Plant Cell 23, 2064–2086. 10.1105/tpc.111.086082 21653195 PMC3160037

[B110] Xiao C. L. Mackenzie S. J. Legard D. E. (2004). Genetic and pathogenic analyses of *Colletotrichum gloeosporioides* isolates from strawberry and noncultivated hosts. Phytopathology 94, 446–453. 10.1094/PHYTO.2004.94.5.446 18943762

[B111] Xie L. Zhang J. Z. Wan Y. Hu D. W. (2010). Identification of *Colletotrichum* spp. isolated from strawberry in Zhejiang province and Shanghai City, China. J. Zhejiang Univ. Sci. B. 11, 61–70. 10.1631/jzus.B0900174 20043353 PMC2801091

[B112] Yen S. K. Chung M. C. Chen P. C. Yen H. E. (2001). Environmental and developmental regulation of the wound-induced cell wall protein WI12 in the halophyte ice plant. Plant Physiol. 127, 517–528. 10.1104/pp.010205 11598226 PMC125087

[B113] Yin Y. Mao X. Yang J. Chen X. Mao F. Xu Y. (2012). dbCAN: a web resource for automated carbohydrate-active enzyme annotation. Nucleic Acids Res. 40, W445–W451. 10.1093/nar/gks479 22645317 PMC3394287

[B114] Zebrowska J. Hortynski J. Cholewa T. Honcz K. (2006). Resistance to *Verticillium dahliae* (Kleb.) in the strawberry breeding lines. Commun. Agric. Appl. Biol. Sci. 71(3 Pt B), 1031–1036. 17390855

[B115] Zhang Q. Y. Zhang L. Q. Song L. L. Duan K. Li N. Wang Y. X.. (2016). The different interactions of *Colletotrichum gloeosporioides* with two strawberry varieties and the involvement of salicylic acid. Hortic. Res. 3:16007. 10.1038/hortres.2016.7 27004126 PMC4793257

[B116] Zipfel C. (2014). Plant pattern-recognition receptors. Trends Immunol. 35, 345–351. 10.1016/j.it.2014.05.004 24946686

