# Supplementary material for: Novel Fungal Pathogenicity and Leaf Defense Strategies Are Revealed by Simultaneous Transcriptome Analysis of Colletotrichum fructicola and Strawberry Infected by This Fungus
Source: Front Plant Sci. 2018 Apr 25;9:434. doi: 10.3389/fpls.2018.00434 (PMC5996897; doi:10.3389/fpls.2018.00434)
Supplement: Supplementary file 3 [file Image1.PDF]

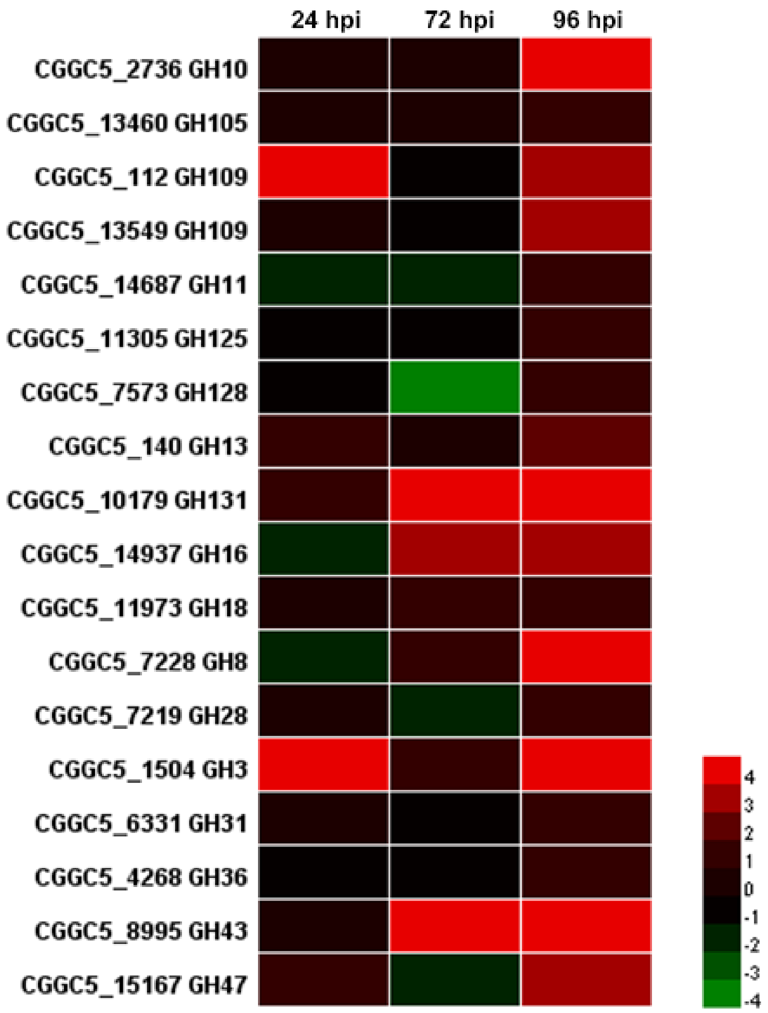

Figure S1. Heat map showing expression levels of GH family of *C. fructicola* during a time course of infection of strawberry. The color bars represent the values of log_2-fold change (infected leaves at 24, 72 and 96 hpi vs mycelium grown in PDA medium) ranging from green (-4) to red (4).
