# Supplementary material for: Novel Fungal Pathogenicity and Leaf Defense Strategies Are Revealed by Simultaneous Transcriptome Analysis of Colletotrichum fructicola and Strawberry Infected by This Fungus
Source: Front Plant Sci. 2018 Apr 25;9:434. doi: 10.3389/fpls.2018.00434 (PMC5996897; doi:10.3389/fpls.2018.00434)
Supplement: Supplementary file 4 [file Image2.PDF]

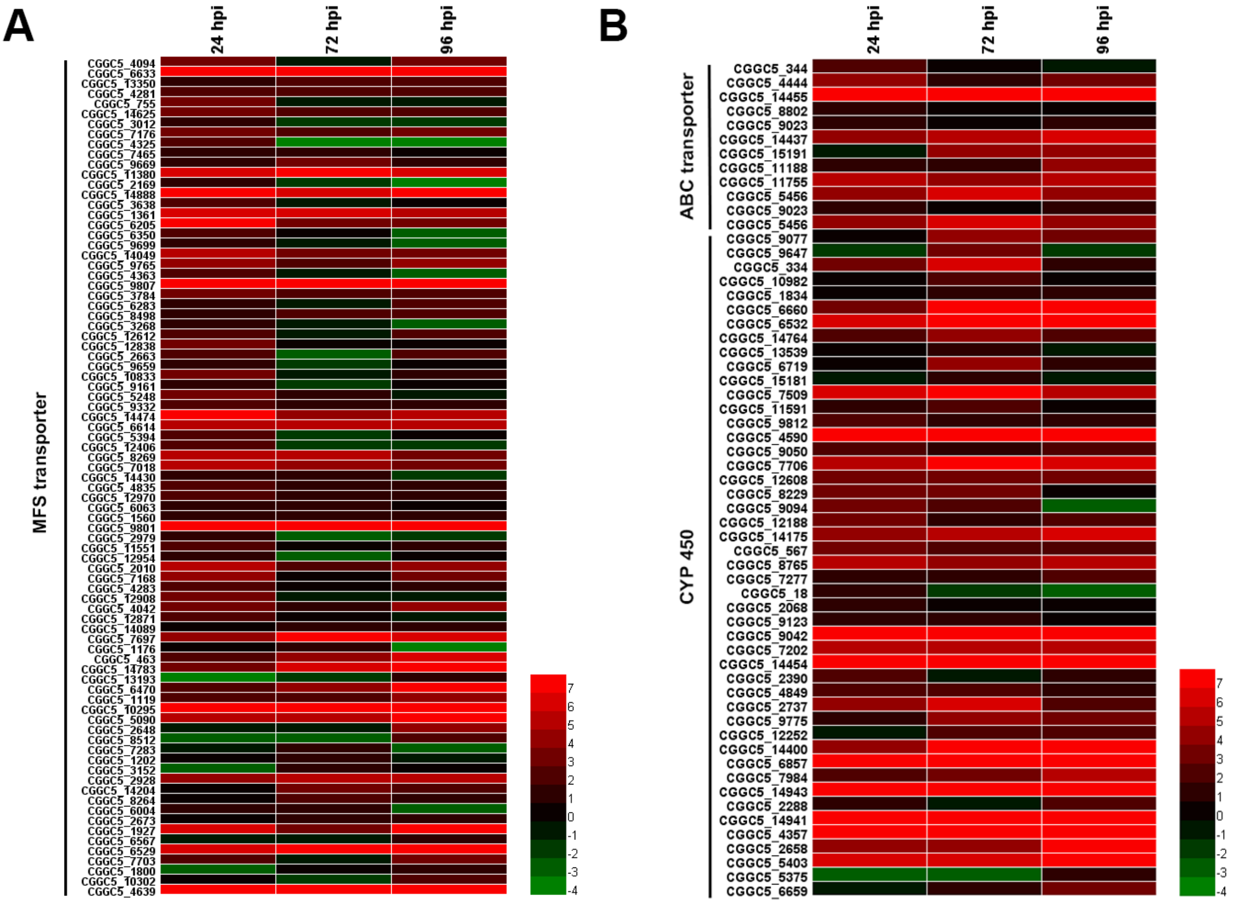

Figure S2. Heat map showing the expression patterns of MFS transporter (A), ABC transporter and CYP450 (B) genes. The color bars represent the values of log2-fold change (infected leaves at 24, 72 and 96 hpi vs. mycelium grown in PDA medium), ranging from green (-4) to red (7)
